# Novel cellular systems unveil mucosal melanoma initiating cells and a role for PI3K/Akt/mTOR pathway in mucosal melanoma fitness

**DOI:** 10.1186/s12967-023-04784-2

**Published:** 2024-01-08

**Authors:** Matilde Monti, Luisa Benerini Gatta, Mattia Bugatti, Irene Pezzali, Sara Picinoli, Marcello Manfredi, Antonio Lavazza, Virginia Vita Vanella, Veronica De Giorgis, Lucia Zanatta, Francesco Missale, Silvia Lonardi, Benedetta Zanetti, Giovanni Bozzoni, Moris Cadei, Andrea Abate, Barbara Vergani, Piera Balzarini, Simonetta Battocchio, Carla Facco, Mario Turri-Zanoni, Paolo Castelnuovo, Piero Nicolai, Ester Fonsatti, Biagio Eugenio Leone, Emilio Marengo, Sandra Sigala, Roberto Ronca, Michela Perego, Davide Lombardi, William Vermi

**Affiliations:** 1 Department of Molecular and Translational Medicine, University of Brescia, Brescia, Italy; 2 Histocompatibility Laboratory “Vittorio Mero”, Department of Transfusion Medicine, ASST Spedali Civili Di Brescia, Brescia, Italy; 3 Department of Translational Medicine, University of Piemonte Orientale, Novara, Italy; 4 Center for Autoimmune and Allergic Diseases, University of Piemonte Orientale, Novara, Italy; 5 Istituto Zooprofilattico Sperimentale Della Lombardia E Dell’Emilia-Romagna “Bruno Ubertini”, Brescia, Italy; 6 Department of Pathology, Treviso Regional Hospital, Treviso, Italy; 7 Department of Head & Neck Oncology & Surgery Otorhinolaryngology, Antoni Van Leeuwenhoek, Nederlands Kanker Instituut, Amsterdam, The Netherlands; 8 Department of Medicine and Surgery, University of Milano-Bicocca, Monza, Italy; 9 Unit of Pathology, Department of Molecular and Translational Medicine, University of Brescia—“ASST Spedali Civili Di Brescia”, Brescia, Italy; 10 Unit of Pathology, Department of Medicine and Surgery, ASST Sette-Laghi, University of Insubria, Varese, Italy; 11 Unit of Otorhinolaryngology and Head & Neck Surgery, Department of Biotechnology and Life Sciences, ASST Sette Laghi, University of Insubria, Varese, Italy; 12 Section of Otorhinolaryngology—Head and Neck Surgery, Department of Neurosciences, University of Padova, Padova, Italy; 13 Medical Oncology and Immunotherapy, University Hospital of Siena, Istituto Toscano Tumori, Siena, Italy; 14 Department of Sciences and Technological Innovation, University of Piemonte Orientale, Alessandria, Italy; 15 The Wistar Institute, Philadelphia, PA USA; 16 Unit of Otorhinolaryngology - Head and Neck Surgery, Department of Medical and Surgical Specialties, Radiological Sciences and Public Health, University of Brescia, Brescia, Italy; 17 Department of Pathology and Immunology, Washington University School of Medicine, Saint Louis, MO USA; 18 Department of Molecular and Translational Medicine, Section of Pathology, University of Brescia, P.Le Spedali Civili 1, 25123 Brescia, Italy

**Keywords:** Mucosal melanomas, Cell lines, Melanoma stem cells, MITF, PI3K/AKT/mTOR

## Abstract

**Background:**

Mucosal Melanomas (MM) are highly aggressive neoplasms arising from mucosal melanocytes. Current treatments offer a limited survival benefit for patients with advanced MM; moreover, the lack of pre-clinical cellular systems has significantly limited the understanding of their immunobiology.

**Methods:**

Five novel cell lines were obtained from patient-derived biopsies of MM arising in the sino-nasal mucosa and designated as SN-MM1-5. The morphology, ultrastructure and melanocytic identity of SN-MM cell lines were validated by transmission electron microscopy and immunohistochemistry. Moreover, in vivo tumorigenicity of SN-MM1-5 was tested by subcutaneous injection in NOD/SCID mice. Molecular characterization of SN-MM cell lines was performed by a mass-spectrometry proteomic approach, and their sensitivity to PI3K chemical inhibitor LY294002 was validated by Akt activation, measured by pAkt(Ser473) and pAkt(Thr308) in immunoblots, and MTS assay.

**Results:**

This study reports the validation and functional characterization of five newly generated SN-MM cell lines. Compared to the normal counterpart, the proteomic profile of SN-MM is consistent with transformed melanocytes showing a heterogeneous degree of melanocytic differentiation and activation of cancer-related pathways. All SN-MM cell lines resulted tumorigenic in vivo and display recurrent structural variants according to aCGH analysis. Of relevance, the microscopic analysis of the corresponding xenotransplants allowed the identification of clusters of MITF-/CDH1-/CDH2 + /ZEB1 + /CD271 + cells, supporting the existence of melanoma-initiating cells also in MM, as confirmed in clinical samples. In vitro, SN-MM cell lines were sensitive to cisplatin, but not to temozolomide. Moreover, the proteomic analysis of SN-MM cell lines revealed that RICTOR, a subunit of mTORC2 complex, is the most significantly activated upstream regulator, suggesting a relevant role for the PI3K-Akt-mTOR pathway in these neoplasms. Consistently, phosphorylation of NDRG1 and Akt activation was observed in SN-MM, the latter being constitutive and sustained by PTEN loss in SN-MM2 and SN-MM3. The cell viability impairment induced by LY294002 confirmed a functional role for the PI3K-Akt-mTOR pathway in SN-MM cell lines.

**Conclusions:**

Overall, these novel and unique cellular systems represent relevant experimental tools for a better understanding of the biology of these neoplasms and, as an extension, to MM from other sites.

**Supplementary Information:**

The online version contains supplementary material available at 10.1186/s12967-023-04784-2.

## Background

Mucosal melanoma (MM) is a highly aggressive and rare form of melanoma arising from melanocytes at any mucosal surface [1]. Due to their hidden sites of origin and the lack of early and specific symptoms, these tumors are often late diagnosed [2, 3]. The majority of the patients undergo surgical resection as a first-line treatment, followed by adjuvant radiotherapy to reduce loco-regional recurrence; however, this approach results in a negligible clinical benefit [3–5]. Local recurrence and distant metastasis are common and associated with a very poor outcome in terms of overall survival rate [2, 6], and the mechanisms leading to this aggressive clinical behavior are only partially understood.

Compared to cutaneous melanoma (CM), MM represent a different entity also in terms of biological features. A diverse cell of origin (mucosal vs cutaneous melanocytes) and a lack of UV-exposure are associated with a distinct molecular profile. Moreover, limited data are available on the existence of MM precursor lesions as well as on their molecular hubs sustaining the progression trajectories [7]. MM rarely show *BRAF V600* mutations [8, 9], thus ruling out treatment with BRAF inhibitors (BRAFi) for the majority of MM patients. Among MM drivers, somatic mutations of *NRAS* and the *TERT* promoter are reported, as well as mutually exclusive loss of function mutation of the tumor suppressors *NF1* and *SPRED1*, the latter co-occurring with activating *KIT* mutations [10–12]. Recent studies indicate that MM have significant genomic instability and display structural variants including amplifications (e.g., *CDK4*, *MDM2*, *TERT*, and *cyclin D1*), deletions (e.g., *CDKN2A/B*, *PTEN* and *TP53*) and fusion genes [10, 13, 14] displaying a site-specific variation [13, 15]. As we reported on a retrospective cohort, many MM cases show enhanced expression of pAkt and pErk, suggesting the activation of both PI3K/Akt/mTOR and RAS/MAPK pathways [16]; but the molecular basis of this activation remains unknown.

A fraction of MM cases might obtain clinical benefits from immunotherapy [17]. In recent years, the role of immune checkpoint inhibitors (ICI) has been evaluated in several monocentric and multicentric retrospective studies [18–20] and clinical trials [21, 22]. To date, ICIs represent the front-line therapy for patients with unresectable advanced or metastatic MM; however, outcomes to this approach remain poor compared to CM [23–30]. The tumor mutational burden (TMB) is substantially low in MM [14, 31] and is more comparable to poorly immunogenic cancers not associated with exposure to mutagens [31]. These genomic and immunogenic differences might explain the lower response rate of MM to ICI blockade [32].

This set of data, although of potential relevance, offers a still limited support to decision making for the clinical management of these patients. Limited understanding of this rare form of cancer is due to the lack of pre-clinical models for genetic and functional studies. Dogs are frequently affected by aggressive spontaneous mucosal melanomas of the oral cavity, providing a unique but still invalidated working model for their human counterparts [33–35].

A better understanding of the immunobiology of MM may benefit from novel cellular models of human origin. Starting from patient-derived fresh biopsies of MM arising in the sinonasal tract, we could generate and successfully propagate five novel cell lines (SN-MM1 to 5). Sinonasal tract MM are among the most frequent MM and on endoscopy they present as expansile or polypoid lesions with different degrees of pigmentation. Patients suffer from nasal obstruction and discharge, epistaxis, facial pain, olfaction disturbance [1]. Additional signs and symptoms (exophthalmos, visual disturbance, headache), suggesting intraorbital and intracranial extension, are in keeping with the heterogeneity of growth patterns (i.e., superficial spreading over the mucosal and submucosal lining of the sinonasal tract *versus* deep infiltration) and multifocality [1, 36]. SN-MM1 to 5 display a melanocytic identity and tumorigenic potential. Moreover, their proteomic analysis revealed activation of cancer-associated pathways involved in cell transformation and cancer progression, including PI3K/Akt/mTOR pathway; as a proof of concept, we could also demonstrate a role for this pathway in MM cell fitness. Finally, microscopic analysis of derived mouse xenograft identified a subpopulation of MM cells coherent with melanoma-initiating cells.

## Methods

### Human tumor biopsies

Human tissues were obtained from surgical specimens from patients undergoing surgery with radical or palliative intent. All samples included in this study were obtained under the approval of the institutional Ethics Board of ASST Spedali Civili of Brescia (IRB code: NP 2066/2015) and ASST Sette Laghi of Varese (IRB code: NP 33025/2015), Italy. Informed consent was obtained from all patients for the manipulation of human tissue and cell culture. Diagnosis of sinonasal mucosal melanoma was performed according to the American Joint Committee on Cancer/Union for International Cancer control staging system for mucosal melanoma of the upper aerodigestive tract [37]. Demographics (gender, age) and selected oncologic informations (site of origin and recurrence, TNM classification, treatment strategy and outcome) [2, 36, 38] are summarized in Additional file 6: Table S1. Briefly, 80% of patients were female and older than 60 years. All patients were in advanced stage disease (T3-T4) and underwent endoscopic endonasal resection followed by radiation therapy (4/5). All patients were died of disease.

### Generation of primary SN-MM cell lines

SN-MM cell lines were generated from fresh surgically collected tumor samples. Surgical specimens were placed in RPMI 1640 medium (cat.n. F1215, Biochrom, Berlin, Germany) supplemented with 20% Fetal Bovine Serum (FBS) (cat.n. S0115, Biochrom), 1% Penicillin*/*Streptomycin (cat.n. 15070-063, Thermo Fisher Scientific, Waltham, MA, USA) on ice. Samples were finely minced with a scalpel in a 100 mm cell culture dish in sterile conditions. After mechanical dissociation, samples were transferred in 15 mL sterile tube with RPMI 1640 medium supplemented with 10% FBS, 2 mM Glutammine (cat.n. 25030149, Thermo Fisher Scientific), 0.5% Penicillin*/*Streptomycin, and 200 U/mL Collagenase Type II (cat.n. LS004174, Worthington, Biochemical Corporation, USA). Samples were incubated at 37 ℃ in a swinging water bath for 3 h and vortexed every 30 min. The enzymatic digestion was stopped by adding DPBS (cat.n. 14190144, Thermo Fisher Scientific) and cell suspension was filtered through 40 µm cell strainer and centrifuged at 10 min at 300 *g*. Red blood cells lysis was achieved with 1X RBC Lysis Buffer (cat.n. 420301, Biolegend, San Diego, CA, USA) following manufacturer’s instructions. Resulting cells were resuspended in RPMI 1640 medium supplemented with 10% FBS, 2 mM Glutammine, 0.5% Penicillin*/*Streptomycin and 100 µg/mL Primocin (InvivoGen, San Diego, CA, USA), seeded in T25 culture flask at density of 5 × 10^5^ cells/mL and maintained at 37 ℃ in a 5% CO_2_ humidified incubator. The following day, cell culture medium was replaced with fresh medium. When adherent cells reached 80% of confluence, cells were detached using the Tryple Express Enzyme (cat.n. 12604013, Thermo Fisher Scientific) and sub-cultured at 1:2 or 1:3 ratio. Cell lines were named SN-MM1-5 and sub-cultured for at least 5–10 times before further analysis.

SN-MM F1 cell lines were generated from fresh cell-derived xenografts (CDX) samples harvested from mice, as described above.

### Cell culture

The SN-MM cell lines were culture with complete RPMI medium consisting of RPMI 1640 supplemented with 10% FBS, 2 mM Glutammine, 0.5% Penicillin*/*Streptomycin. Normal human epidermal melanocytes (NHEM) M2 (cat.n. C-12400, PromoCell, Heidelberg, Germany) and M3 (cat.n. C-12413, PromoCell) were cultured in Melanocytes Growth Medium M2 (cat.n. C-24300, PromoCell) and M3 (cat.n. C-24310B, PromoCell), respectively, following manufacturer’s instructions. Mycoplasma contamination were excluded by routinely testing with Universal Mycoplasma detection kit (cat. N. 30-1012K ATCC, Manassas, VA) according to manufacturer’s suggestions.

### Cell block preparation

For cell-block preparation, at least 1 × 10^6^ cells were harvested and processed as follows. Cell suspensions of SN-MM cell lines were centrifuged for 10 min at 3,000 rpm. A solution of plasma (100 mL, kindly provided by Centro Trasfusionale, ASST Spedali Civili) and HemosIL RecombiPlasTin 2G (200 mL, Instrumentation Laboratory; cat. no. 0020003050; 1:2) were added to cell pellets, mixed until the formation of a clot, then placed into a labeled cassette (Bio-Optica; cat. no. 07-7350). The samples were fixed in 10% formalin (Bio-Optica; cat. no. 05-K01004) for 1 h followed by paraffin inclusion.

### Histology and Immunohistochemistry

Formalin fixed and paraffin embedded (FFPE) tumor biopsies, xenografted tumors and cell-block sections were used for histology and immunohistochemistry (IHC).

Haematoxylin–eosin (H&E) staining was performed using standard protocols on 4 µm sections. Histologic evaluation of H&E-stained sections of parental tumor samples, cell-blocks and xenografts was performed by two expert pathologists. Four-micron thick tissue sections were used for immunohistochemical staining, heat mediated antigen retrieval was performed in microwave oven and endogenous peroxidase activity was quenched using 0.3% hydrogen peroxide diluted with methanol during rehydration. After washing with Tris-Buffered Saline (TBS) solution, slides were incubated with primary antibody for 1 h at room temperature and revealed by incubation with horseradish-peroxidase polymer Novolink Polymer Detection System (cat.n. RE7159, Leica Biosystems, Wetzlar, Germany), Envision + System-HRP Labelled Polymer Anti-mouse (cat.n. K4001, Dako) or Anti-Rabbit (cat.n. K4003, Dako), followed by 3,3ʹ-diaminobenzidine as chromogen. Sections were counterstained with Mayer’s haematoxylin (Bioptica, Milano, Italy). Primary antibody used are listed in Additional file 6: Table S2.

IHC evaluation of melanocytic markers was performed using a four-tiered scoring system (score 0 = 0% of positive cells; score 1 = 1–10% of positive cells; score 2 = 10–50% of positive cells; score 3 > 50% positive cells).

For double sequential immunostains, the first reaction is deleted after first chromogen de-stain and stripping. Anti-CDH1 was used for the first immune reaction, revealed using Novolink Polymer (Leica) and developed in 3-amino-9-ethylcarbazole chromogen (AEC), counterstained with hematoxylin and cover-slipped using gelatin. Subsequently, the slides were digitally scanned, using Aperio Scanscope CS (Leica Microsystems). After cover slip removal, AEC was washed out and the slides were eluted using a 2-Mercaptoethanol/SDS solution (20 mL 10% w/v SDS with 12.5 mL 0.5 M Tris–HCl, pH6.8, 67.5 mL distilled water and 0.8 mL 2-ME). Slides were subsequently incubated in this solution in a water-bath pre-heated at 56 ℃ for 30 min. Sections were washed for 1 h in distilled water. After unmasking in microwave, anti-ZEB1, was revealed using Novolink Polymer (Leica) and AEC CD271 was revealed using Mach 4 MR-AP and Ferangi blue as chromogen and slides were counterstained with hematoxylin, cover-slipped and digitally scanned. The subsequent immunostains for anti-MITF1 was developed analogously. The digital slides were processed using ImageScope. Slides were synchronized and corresponding tissue regions were analysed.

### Immunofluorescence

SN-MM cells (3 × 10^5 ^cells) were seeded in Nunc^™^ Lab-Tek^™^ II Chamber Slide^™^ System (cat.n. 154453 Thermo Fisher Scientific). Cells were fixed in ethanol 95% and were used for immunofluorescence (IF) staining. Heat mediated antigen retrieval was performed in microwave oven. After washing with TBS solution, slides were blocked with TRIS plus 5% BSA solution for 30 min. Then, slides were incubated with primary antibody for 1 h at room temperature and revealed by incubation with horseradish-peroxidase polymer Novolink Polymer Detection System (cat.n. RE7159, Leica Biosystems, Wetzlar, Germany), followed by Alexa Fluor TM 488 tyramide reagent (cat. n. B40953, Invitrogen, Waltham, MA, USA) and cyanine TM 555 tyramide reagent (cat. n. 96020, Biotium, Fremont, CA, USA). Fluorescence Mounting Medium (cat. n. S3023, Dako) was used. Primary antibody used are listed in Additional file 6: Table S2.

The slides were digitalized using Axioscan7 (Zeiss) and processed using Zen Blue software (Zeiss).

### Transmission electron microscopy

2 × 10^6^ cells were collected and washed once with DPBS. Cell pellets were fixed with 2.5% glutaraldehyde (Sigma-Aldrich, Cat. N. G5882) in Sorensen’s Phosphate Buffer pH 7.2 and kept at 4 ℃ for at least 2 h. After fixation, pellets were washed three times with Sorensen’s phosphate buffer for and post-fixed for at least 2 h with 1% Osmium Tetroxide (Electron Microscopy Sciences, Cat.#19134). Samples were washed in distilled water and dehydrated in progressive ethanol concentration (50%, 70%, 90% and 100%) for 15 min at each step. Cell pellets were soaked in acetone for 15 min and then transferred in a 1:1 mixture of acetone and Epoxidic resin for at least 3 h. Embedding was accomplished in Epoxidic resin. Semithin (1 µm-thick) sections cut with glass sharp blade using an ultramicrotome (Ultracut E- Leica, Microsystems S.r.l., Milan, Italy) were stained with toluidine blue and examined by light microscopy. For ultrastructural analysis, 80 nm-thick sections, cut with diamond glass were obtained from selected areas and were collected on 200 meshes formvar coated copper grids, double stained with uranyl acetate (Electron Microscopy Sciences Cat. N. 22409) and lead citrate (Electron Microscopy Sciences Cat. N. 22410) and examined using FEI Tecnai G2 Spirit or CM12 TEM transmission electron microscopes (FEI Instrumentation Company, Hillsboro, Oregon, USA) operating at 85kV. Photographs were taken using a Veleta integrated Digital Camera (Olympus Soft Imaging Solutions, Munster, Germany).

### DNA isolation

Genomic DNA was extracted from SN-MM cell lines using QIAamp DNA Blood Mini Kit (cat. n. 51104, Qiagen, Redwood City, CA, USA) according to manufacturer's protocol. Automated DNA Extraction was performed on the FFPE tissues using Maxwell® RSC DNA FFPE Kit (cat. n. AS1450, Promega, Madison, WI, USA) according to manufacturer’s protocol.

### Array comparative genomic hybridisation (aCGH)

aCGH experiments were performed on gDNA from SN-MM cell lines. To avoid false negative results due to the sensitivity of this assay, only samples displaying a tumor cells content greater than 50% were analyzed. The genomic DNA was prepared and purified to a standard where the A260/A280 ratio exceeded 1.8 and A260/A230 ratio exceeded 1.5. The amount of DNA requested to perform the test was 1.0 µg. Samples and references were labelled using the CytoSure Genomic DNA Labelling Kit (Oxford Gene Technology). The CytoSure Array 8 × 60 k CGH were scanned by Agilent Surescan C scanner with 2 μm resolution; features were extracted with Feature Extraction software and log2 ratio data were imported and analyzed by Cytosure Interpret Software 4.11.36 (Oxford Gene Technology) for the identification of copy number variation (CNV). Copy number changes below 5Mb was not reported unless affecting a gene/region relevant to cancer disease.

### Fluorescence in situ hybridization (FISH)

Interphasic FISH Test *IGH-MYC-CEP8* and FISH Test *CCND1/CEP11* were performed on 4 μm FFPE (Formalin Fixed Paraffin Embedded), probes *IGH-MYC-CEP8* were provided by Abbott Molecular (Des Plaines, USA), while *CCND1/CEP11* by Zytovision GmbH (Bremerhaven, Germany) and were used following the manufacturer’s suggested protocol. For each case, a minimum of 50 nuclei were observed using the Leica DM6000B System (Leica Microsystems, Buccinasco, MI, Italy). A total of 100 cells were evaluated for signal pattern for all probes. Nuclei with at least 15% with rearranged patterns were considered positive.

### Sanger Sequencing

PCR was performed to amplify NRAS exon 2 using the specific primers (NRAS_Forward: 5ʹCAACAGGTTCTTGCTGGTGT3ʹ; NRAS_Reverse: 5ʹCCTCACCTCTATGGTGGGAT3ʹ). The PCR products were purified using the Amicon Ultra 0.5 mL Centrifugal Filters 30 K (cat. n. UFC5030BK, Millipore, Burlington, MA, USA), according to the manufacturer’s instructions, and sequenced in both directions using the BigDye^™^ Terminator v3.1 Cycle Sequencing Kit (cat. n. 4337455; Applied Biosystems). The excess of fluorescent terminator dye was removed using Performa^®^ DTR Gel Filtration Cartridges (cat. n. 4408228, Edge Bio Systems, San Jose, CA, USA). The samples were resuspended in formamide, denatured at 95 ℃ for 3 min and processed through SeqStudio™ Genetic Analyzer (ThermoFisher Scientific).

### Flow cytometry

SN-MM cells were detached using the Tryple Express Enzyme (cat.n. 12604013, Thermo Fisher Scientific), washed in 1 mL cold DPBS w/o proteins and stained using Live/Dead Fixable Red Dead Cell Stain Kit (cat. n. L23102, Life Technologies) and anti-CD271 (clone REA844) PE-conjugated antibody (cat.n. 130-112-601, Miltenyi Biotec, Bergisch Gladbach, Germany) following manufacturer’s instructions. Unstained cells were used as negative controls. Results were reported as the percentage of positive cells after gating on single live cells. Samples were acquired with MACS Quant^®^ Analyzer 16 (Miltenyi Biotec) and results were analyzed by FlowJo X software v10.8 (Tree Star Inc, Wilmington, NC, USA).

### MTS assay

15 × 10^3^ cells were plated in flat bottom 96-well plates in 125 µL complete RPMI medium. The LY294002 (1–100 µM; cat.n. s1105, Selleckchem, Houston, TX, USA) was used and cells were cultured for 24 h, 48 h and 72 h. The DMSO was used as vehicle control at the final concentration of 0.2%. The cisplatin (0.625–20 µM; cat.n. s1166, Selleckchem) and temozolomide (3.125–200 µM; cat.n. s1237, Selleckchem) were used and cells were cultured for 72 h or 120 h. The 0.1% DMF and 0.4% DMSO were used as vehicle controls, respectively. Cell proliferation was evaluated using the CellTiter 96^®^ AQueous One Solution Cell Proliferation Assay (cat.n. G3580, Promega), according to the manufacturer's instructions. Absorbance was determined at 490 nm with EnSight^™^ multimode plate reader (PerkinElmer, Waltham, MA, USA). The sigmoidal concentration–response function was applied to calculate the IC50 values.

### Western blotting

1–1.5 × 10^5 ^cells were seeded in 12-well plates in 1 mL complete RPMI medium. After eight hours from seeding, cells were starved in RPMI medium with 1% FBS overnight (o/n), then treated with 50 µM LY294002 for 4 h and 24 h. Cells were lysed in RIPA lysis buffer (cat.n. 89900, Thermo Fisher Scientific) supplemented with Protease Inhibitor Cocktail (cat.n. 78440, Sigma-Aldrich) and incubated on ice for 20 min. Protein concentration was determined by Bradford assay and 20 µg of total proteins were loaded on 4–12% NuPAGE^®^ Bis–Tris Mini Gels (cat.n. NP0335, Invitrogen) under reducing condition and transferred onto a PVDF membrane (cat.n. LC2007, Invitrogen). Membranes were blocked with 5% milk (cat.n. 22012, Biotium, Fremont, CA) in TBS-T (TBS with 0.05% Tween 20; cat.n. 28360, Invitrogen) for 1 h at room temperature. Primary antibodies were incubated o/n at 4 ℃ in TBS-T with 5% BSA (cat.n. A3059, Sigma-Aldrich). Primary antibodies are listed in Additional file 6: Table S2. The anti-Rabbit (cat.n. 31460, Thermo Fisher Scientific) or the anti-mouse (cat.n. 7076, CST) secondary antibodies conjugated with horseradish peroxidase were incubated for 1 h at room temperature. Detection was performed using the SuperSignal^™^ West Pico Chemiluminescent Substrate (cat.n. 34577, Thermo Fisher Scientific) and visualized by autoradiography.

### Tumorigenic assay

6 weeks old NOD/SCID mice (Envigo, Udine, Italy), were subcutaneously injected in the dorsolateral right flank with 8 × 10^6^ cells in 1:1 200 µL mixture of PBS and Matrigel (Cultrex BME, R&D Systems). Mice were euthanized when tumors 300 mm^3^ volume and tumors were harvested and paraffin included for histological examination. Animal experiments were approved by the local animal ethics committee and were performed in accordance with national guidelines and regulations. Procedures involving animals and their care are conformed with institutional guidelines that comply with national and international laws and policies (EEC Council Directive 86/609, OJ L 358, 12 December 1987) and with “ARRIVE” guidelines (Animals in Research Reporting In Vivo Experiments).

### Proteomic analysis

4 × 10^4^ M3-NHEM and 1–1.5 × 10^5^ SN-MM cells were seeded in 12-well plates in 1 mL complete medium. Cells were collected, washed, and lysed, as described in the *Western blotting Materials and Methods Section*. After cell lysis, the proteins were digested with trypsin. 40 µg of protein were reduced in 25 µL of 100 mM NH4HCO3 with 2.5 μL of 200 mM DTT (Sigma) at 60 ℃ for 45 min and next alkylated with 10 μL 200 mM iodoacetamide (Sigma) for 1 h at RT in dark conditions. Iodoacetamide excess was removed by the addition of 200 mM DTT. The digests were dried by Speed Vacuum and then desalted [40, 41].

Trypsin-digested sample proteins were analyzed with a micro-LC (Eksigent Technologies, Dublin, CA, USA) system coupled with a 5600 + TripleTOF system (Sciex, Concord, ON, Canada) equipped with DuoSpray Ion Source. Stationary phase was a Halo C18 column (0.5 × 100 mm, 2.7 µm; Eksigent Technologies, Dublin, CA, USA). Mobile phase was a mixture of 0.1% (v/v) formic acid in water (A) and 0.1% (v/v) formic acid in acetonitrile (B), eluting at a flowrate of 15.0 µL min − 1 at an increasing concentration of solvent B from 2 to 40% in 30 min. For identification purposes the samples were subjected to a data dependent acquisition (DDA) while the quantification was performed through a data independent analysis (DIA) approach. The DDA files were searched using Protein Pilot software v4.2 (Sciex, Concord, ON, Canada) and Mascot v2.4 (Matrix Science Inc., Boston, MA, USA) using trypsin as enzyme, with 2 missed cleavages, a search tolerance of 50 ppm for the peptide mass tolerance, and 0.1 Da for the MS/MS tolerance. The UniProt Swiss-Prot reviewed database containing human proteins (version 01/02/2018, containing 42271 sequence entries), with a false discovery rate fixed at 1%.

Label-free quantification was carried out with PeakView 2.0 and MarkerView 1.2 (Sciex, Concord, ON, Canada). Six peptides per protein and six transitions per peptide were extracted from the DIA files. Shared peptides were excluded as well as peptides with modifications. Peptides with FDR lower than 1.0% were exported in MarkerView for the t-test (p-value < 0.05 and fold change > 1.3). Multivariate statistical analysis was performed through MetaboAnalyst 5.0 (www.metaboanalyst.org). Cluster analysis was done by k-means after assessing the best number of clusters maximizing the average silhouette width. Bioinformatic analysis was carried out using Ingenuity Pathways Analysis (IPA) software (Qiagen), Gene Set Enrichment Analysis v7.4 (GSEA), DAVID tools (https://david.ncifcrf.gov), STRING software (https://string-db.org) [39] and R version 4.2.0 (R Foundation for Statistical Computing, Vienna, Austria) [40] was used for statistical analysis including the use of the following packages: (“ComplexHeatmap” [41], “ggplot2” [42], “ggpubr” [43], “PCAtools” [44], “factoextra” [45]. The mass spectrometry proteomics data have been deposited to the ProteomeXchange Consortium via the PRIDE partner repository with the dataset identifier PXD037551.

## Results

### Morphology and phenotype of SN-MM cells are consistent with a melanocytic identity.

**Fig. 1:**
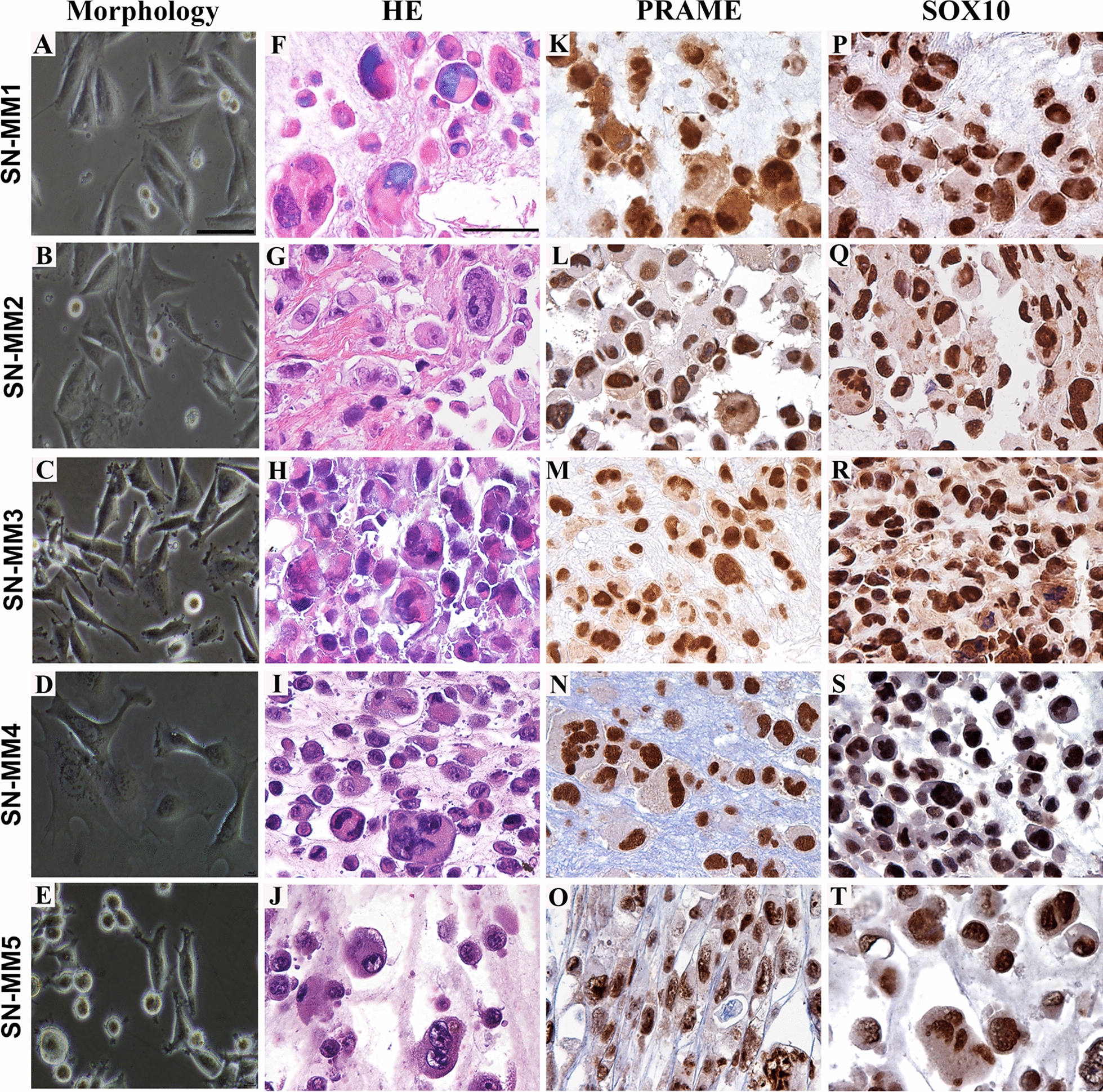
Morphology and phenotype of SN-MM cell lines. Representative images of SN-MM cell lines are from a phase-contrast microscopy (**A**–**E**) or from cell-block sections of fixed cells (**F**–**T**) and stained as labelled. SN-MM cells display an epithelioid or spindle cell morphology with pleomorphic features (**A**–**J**) and strongly express Preferentially expressed Antigen in Melanoma (PRAME) (**K–O**) and SRY-box transcription factor 10 (SOX10) (**P–T**). Sections are counterstained with haematoxylin. Magnification 10X, scale bar 500 µm (**A**–**E**); magnification 400X, scale bar 50 µm (**F-T**)

We received 37 clinical samples from sinonasal melanoma. We successfully established five cell lines (13.5%) and we named them SN-MM1-5. After five sub-culture passages, the fibroblastic cells decreased and disappeared, giving rise to 100% tumor cell population. Cells were maintained in culture for more than thirty passages. Validation of their melanoma identity was obtained based on their morphology and phenotype (Fig. 1 and Additional file 7: Figure S1-S2). In culture, adherent tumor cells, growing in a monolayer, were heterogeneous in size and morphology. SN-MM1, SN-MM3 and SN-MM4 show an epithelioid morphology, instead, SN-MM2 and SN-MM5 were mostly spindled (Fig. 1A–E). SN-MM5 also showed cells growing in suspension as single cells or spherical clusters (Fig. 1E). SN-MM cell lines were propagated for a minimum of ten passages before phenotypical validation by histology. Thereafter, as evaluated on cell-block sections, SN-MM cells displayed atypical morphology on H&E (Fig. 1F–J) and a high proliferation index, as measured by Ki67 (mean: 56.6% SN-MM1; 67.9% SN-MM2; 36.4% SN-MM3; 43.2% SN-MM4; 84.5% SN-MM5) and Histone H3 immunostaining (mean: 4.3% SN-MM1; 2.5% SN-MM2; 1.3% SN-MM3; 0.6% SN-MM4; 5.6% SN-MM5) (Additional file 7: Figure S3A–C). Of note, SN-MM cell lines were strongly positive for SRY-box transcription factor 10 (SOX10) and preferentially expressed antigen in melanoma (PRAME) (Fig. 1K–T). Moreover, SN-MM cell lines variably retained the expression of classical melanocytic markers, including the microphthalmia-associated transcription factor (MITF), human melanoma black 45 (HMB-45), melanoma antigen recognized by T cells 1 (MART-1), tyrosinase (TYR), and S100 calcium-binding protein B (S100), similarly to corresponding parental tumor biopsies (Additional file 7: Figure S1 and S2). Specifically, SN-MM1, SN-MM2 and SN-MM5 displayed more homogeneous melanocytic phenotypes, whereas SN-MM3 and SN-MM4 lose most melanocytic markers in a significant fraction of cells. Accordingly, the pattern of expression of MART, SOX10 and S100 was confirmed by immunofluorescence on 2D SN-MM cell lines (Additional file 7: Figure S4). No contamination by tumor-associated fibroblasts was observed as documented by negative stain for α-SMA (Additional file 7: Figures S1 and S2).

### Ultrastructure of SN-MM cell lines is coherent with a heterogeneous level of melanocytic differentiation.

In histology, melanin is detected in normal and transformed mucosal melanocytes [46, 47]; accordingly, variable expression of HMB-45 and tyrosinase, required for melanin biosynthesis, has been reported in SN-MM cases, as well [46, 48]. Melanosomes correspond to the ultrastructural hallmark of cells with a full melanocytic differentiation, representing cellular organelles designated for melanin biosynthesis, storage, and transfer [49]. Melanosomes develop through four morphologically distinct stages. The pre-melanosomes (stage I) are identified as very low electron-dense non-pigmented vesicles; stage II immature melanosomes contain thin parallel rods corresponding to the intraluminal proteinaceous fibrils, where melanin granules deposit at and arrange in periodic fibrillar structures with a “zig-zag” or coil pattern (stage III). Finally, melanosomes mature to stage IV, containing an amorphous conglomeration of melanin and appearing as high electron-dense vesicles [49, 50].

**Fig. 2:**
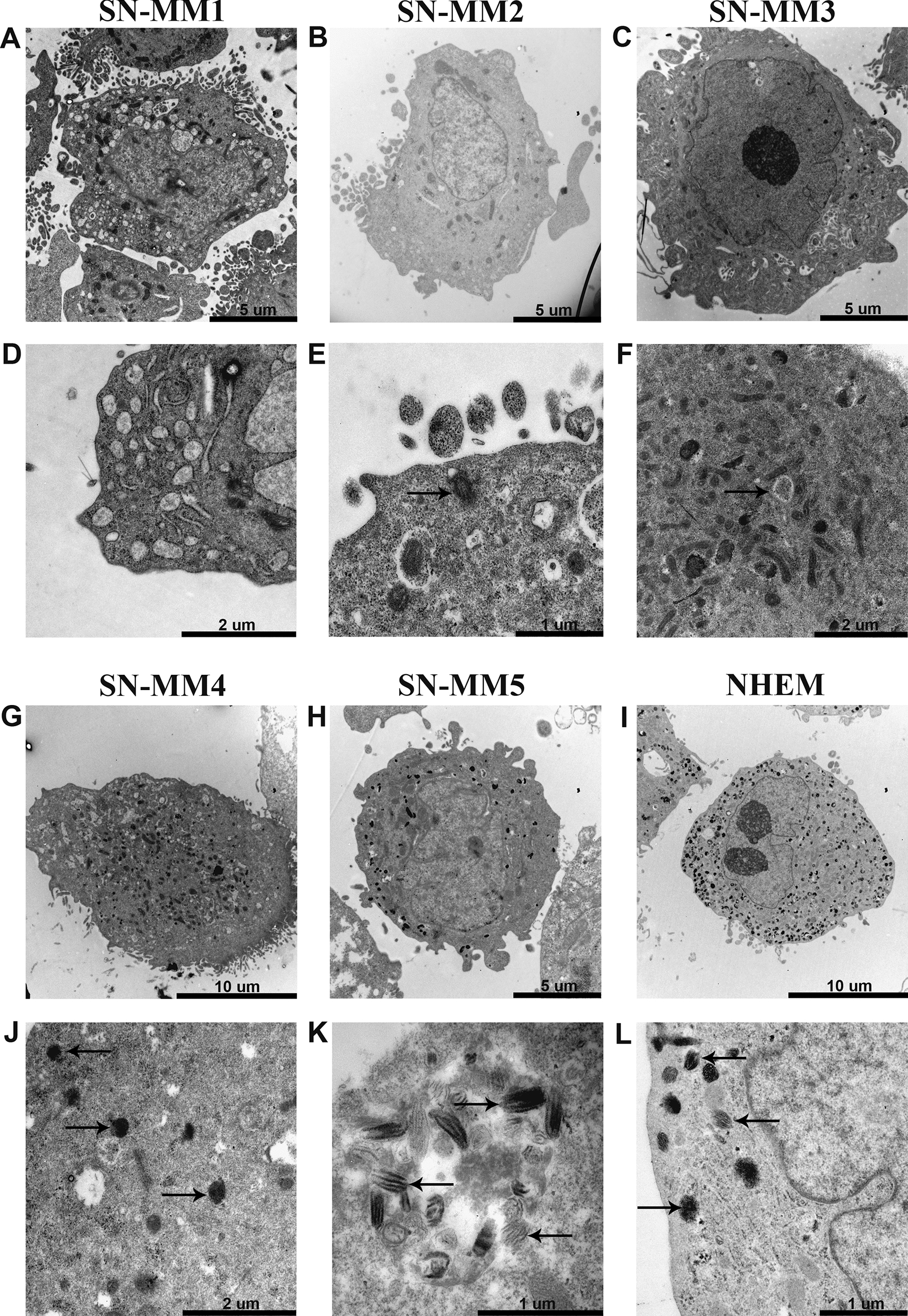
Ultrastructural features of SN-MM cell lines. TEM images show SN-MM cell morphology (**A**–**C**, **G**–**I**) and melanosomes (black arrows) at different stages of development including early (**E**, **F**) and mature (**J**–**L**) melanosomes. A well-developed Golgi apparatus is also shown (**D**). Scale bars are 10 µm (**G, I**), 5 µm (**A**, **B**, **C**, **H**), 2 µm (**D**, **F**, **J**) and 1 µm (**E**, **K**, **L**)

Normal melanocytes (commercial NHEM cells) are characterized by the abundance of mature melanosomes, although heterogeneously distributed among cells, as revealed by TEM analysis (Fig. 2 and Additional file 7: Figure S5). They appear round or polyhedral in shape and show villus-like projections at the cell surface. Their nuclei are large, presenting a definite indentation, and containing large electron dense nucleoli (Additional file 7: Figure S5). According to their differentiation, numerous cytoplasmic melanosomes are detectable, located at the periphery of the cell and in the mitochondria-enriched areas (Fig. 2 and Additional file 7: Figure S5). Melanosomes appear as ovoid or round vesicles, ranging from 0.2 µm to 0.6 µm along the longitudinal axis, delimited by a membrane. SN-MM cells resemble normal melanocytes in morphology. They contain numerous large mitochondria, a well-developed endoplasmic reticulum (ER), and a Golgi apparatus endowed with large cisternae and many vesicles. Based on the presence of a brownish pigmentation of cell pellets, SN-MM cell lines result as melanotic (SN-MM5) or as amelanotic (SN-MM1 to -4) melanomas (Additional file 1). TEM analysis identified melanosomes at various stages of differentiation and heterogeneously distributed (Fig. 2 and Additional file 7: Figure S6). High numbers of melanosomes, at various stages of melanin production, were detected in the cytoplasm of SN-MM5 cells (Fig. 2H, K and Additional file 7: Figure S6I), whereas few and mainly immature melanosomes (stage I-II) were found in SN-MM1, SN-MM2 and SN-MM3 (Fig. 2A–F and Additional file 7: Figure S6A–G); SN-MM4 included rare, pigmented melanosome (stage III-IV) (Fig. 2G, J and Additional file 7: Figure S6H).

Overall, ultrastructure was confirmatory of a melanocytic origin, although with a heterogeneous level of differentiation.

### Recurrent structural variations in chromosome regions containing cancer genes are found in SN-MM cell lines.

**Fig. 3:**
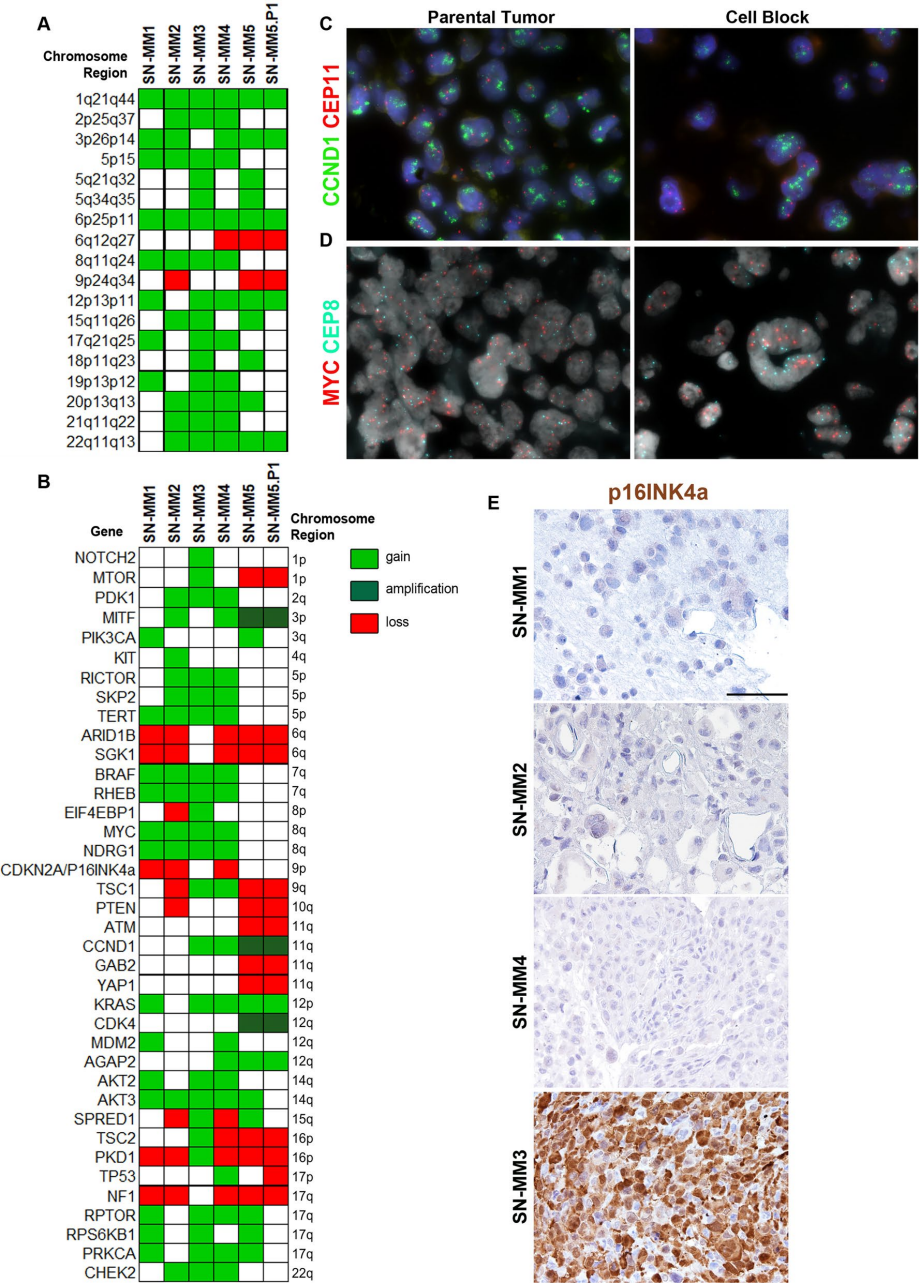
Significant chromosome regions and genes affected by copy number variation (CNV) in SN-MM cell lines. (**A**, **B**) Heatmaps show the chromosome regions recurrently affected by gains or losses (**A**) and significant genes, previously identified as melanoma driver genes or component of PI3K/Akt/mTOR pathway, affected by CNVs (**B**) as detected by aCGH. The green blocks indicate copy number gain/amplification and red blocks indicate copy number loss/deletion, as labeled. (**C**, **D**) FISH analysis of the amplified regions including CCND1 amplification in SN-MM5 (**C**) and MYC amplification in SN-MM3 (**D**) parental tumor biopsies and cell-blocks. (**E**) Immunohistochemistry for p16^INK4a^ on cell blocks from SN-MM samples. Lack of p16^INK4a^ expression is observed in SN-MM1, SN-MM2 and SN-MM4, whereas strong reactivity is observed in SN-MM3. Sections are counterstained with haematoxylin. Magnification 200X; scale bar 100 µm

Complex genomic rearrangements, including focal amplification and losses, are recurrent in MM [14, 31, 51]. We performed aCGH analysis to investigate the genome-wide copy-number variations (CNVs) in SN-MM cell lines. The aCGH profiles show remarkably consistent alterations in SN-MM cell lines, including the gain of chromosome arms 1q, 6p, 8q, and the loss of 6q and 9p chromosome regions (Fig. 3A and Additional file 7: Figure S7**)**, as previously reported in MM [52].

To identify gene candidates in SN-MM transformation and progression, we interrogated the CNV status of known cancer genes with a focus on already well-studied melanoma-genes. Results revealed strong similarities with MM cohorts previously described [10, 13, 14, 53, 54] with many of the genomic rearrangements targeting well-known melanoma-related genes (Fig. 3B). Specifically, SN-MM cell lines harbored recurrent gain of *TERT*, *BRAF*, *MYC*, *KRAS*, *SKP2*, and *MITF* (Fig. 3B). The oncogenic driver *MYC* plays an important role in cell cycle control through Wnt-signaling and contributes to chromosomal instability [55]. Moreover, the tumor suppressors *ARID1B*, *CDKN2A* and *NF1* were commonly affected by chromosomal copy loss (Fig. 3B). The CDKN2A/CDK4/6/CCND1 complex is an essential regulator of cell cycle [56]; CDK4/6/CCND1 leads to the G1-S phase transition, whereas CDKN2A negatively regulates cell cycle progression. Accordingly, *CDK4* and *CCND1* were amplified, while homozygous deletions of *CDKN2A* were found in three out of five SN-MM cell lines (Fig. 3B). Loss of the *CDKN2A* gene, encoding the tumor suppressor proteins p16^INK4a^ and p14^ARF^, is a frequent event driving melanoma progression, including MM [10, 13]. Interestingly, we also observed loss of *PTEN* (SN-MM2 and SN-MM5) and *SPRED1* (SN-MM2 and SN-MM4) in two out of five SN-MM cell lines (Fig. 3B). *PTEN* is a well-established tumor suppressor gene in various human cancers [57] and is the central negative regulator of PI3K/Akt pathway; loss of PTEN leads to increased AKT activation, causing cell proliferation, survival, migration and spreading [58, 59]. Additional findings have demonstrated that PTEN also plays a critical role in DNA damage repair and DNA damage response [60]. The *SPRED1* gene is a tumor suppressor that encodes a negative regulator of MAPK signaling, previously identified as a driver in MM [11]. Specifically, SPRED1 acts by transporting NF1 to the plasma membrane where it inhibits RAS-GTP signaling [61]; therefore, loss of SPRED1 results in increased MAPK pathway activity and increased cell proliferation [11]. Of note, further genomic alterations, even though occurring at lower frequencies, indicated commonality of signaling networks in tumorigenesis, including PI3K/AKT/mTOR, Notch signaling, MAPK pathway, and cell cycle pathways leading to the cancer hallmarks of sustained proliferation and evading growth suppression [54]. Moreover, to exclude a propagation bias, we performed aCGH on early passage SN-MM5 cell line (designated as SN-MM5.P1) *versus* the established SN-MM5 cell line (P28) and found a comparable profile (Fig. 3A-B and Additional file 7: Figure S7). As confirmatory to aCGH analysis, we could demonstrate amplification of *MYC* and *CCND1* by FISH (Fig. 3C, D) as well as loss of p16^INK4a^ expression (Fig. 3E), on cell-block preparation and parental biopsies.

### SN-MM cell lines are tumorigenic in vivo and contain a pool of melanoma-initiating cells.

**Fig. 4:**
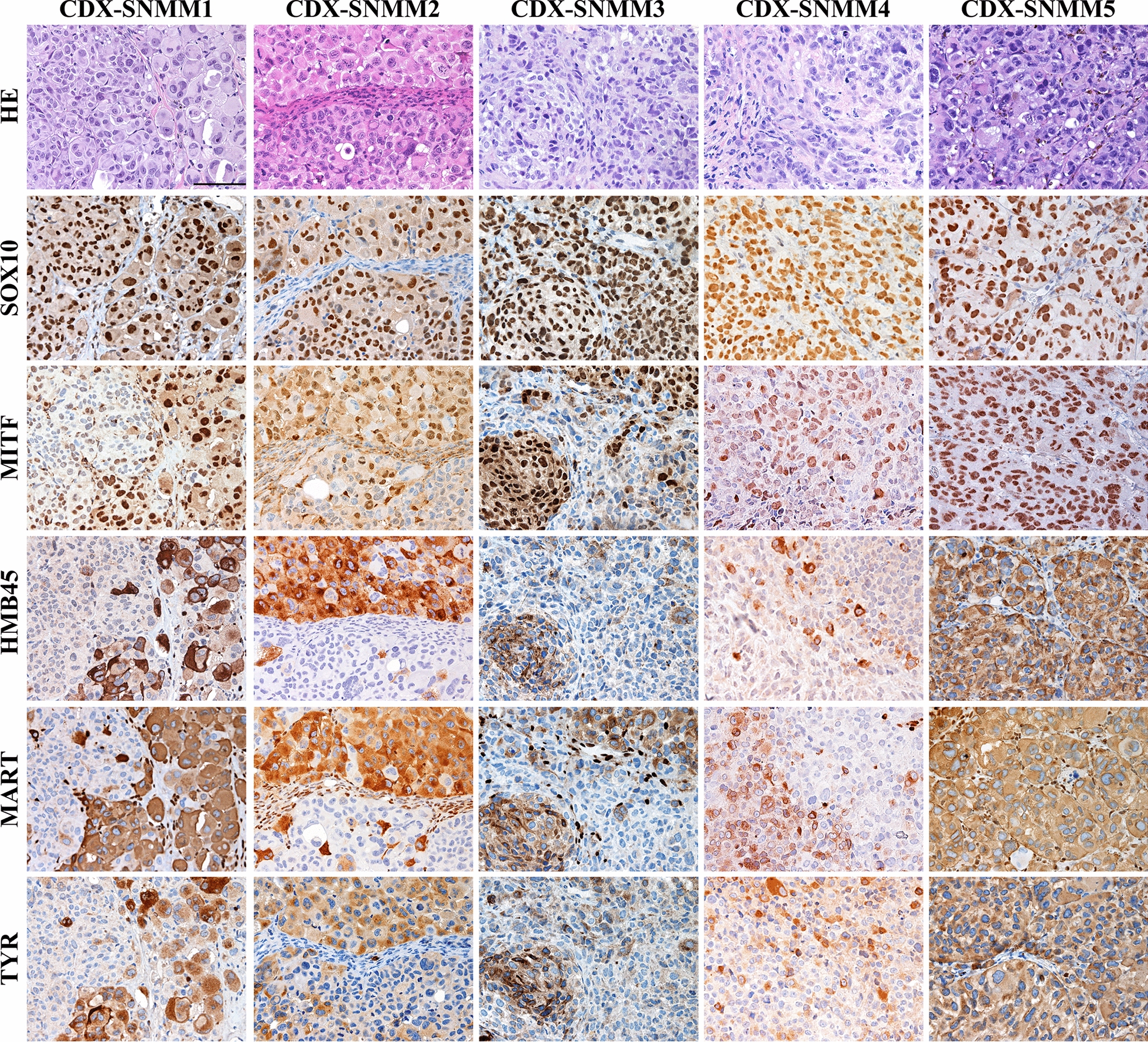
Histology of SN-MM cell-derived xenografts (CDX). Representative images of CDX immunohistochemistry. *First row*: H&E staining of CDX sections showing cellular morphology. *Second to sixth row*: Immunohistochemistry expression of SOX10, MITF, HMB45, MART, and TYROSINASE (TYR) in CDX tissue sections labeled as indicated. Sections are counterstained with haematoxylin. Magnification 200X; scale bar 100 µm

**Fig. 5:**
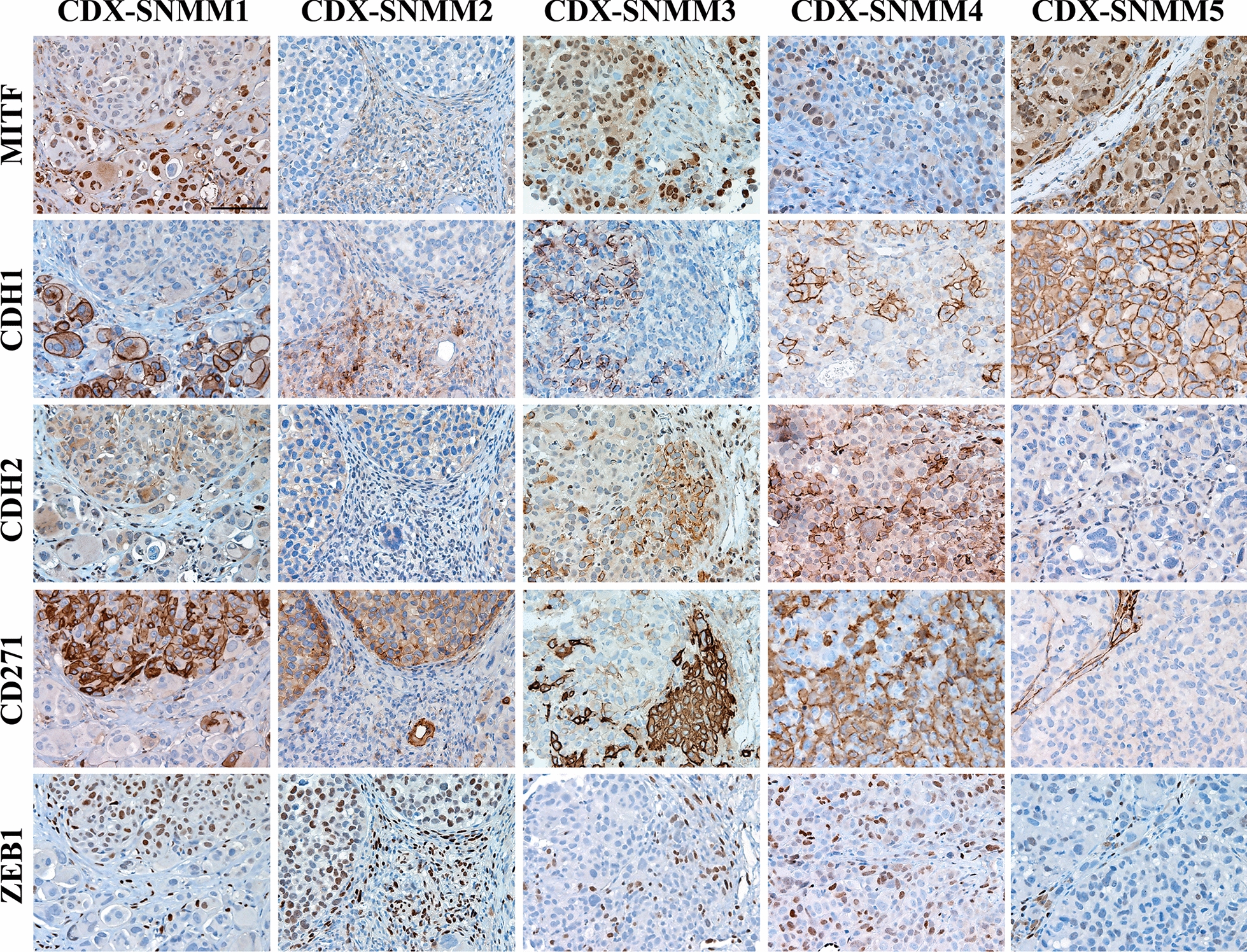
Detection of MIC clusters in SN-MM-CDX. Sections are from CDX and stained as labeled. Representative images of expression of MITF, CDH1, CDH2, CD271, and ZEB1 identify cluster of MIC (MITF^−/low^/CDH1^−^/ZEB1^+^/CD271^+^) in SN-MM-CDX1 to CDX4. Sections are counterstained with haematoxylin. Magnification 200X; scale bar 100 µm

**Fig. 6:**
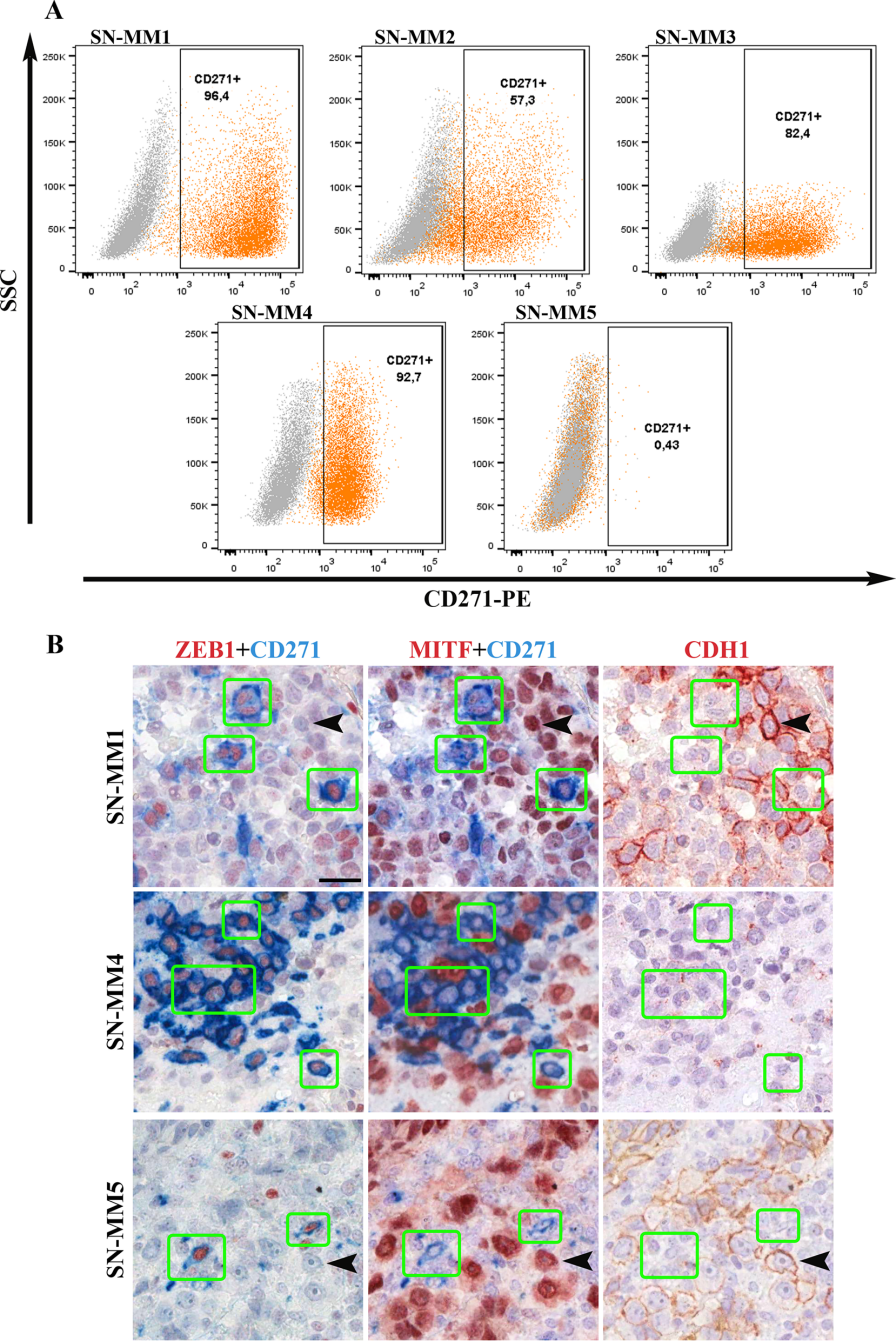
Expression of CD271 on SN-MM cells and co-expression of CD271 and ZEB1 on parental tumors. **A** CD271 flow cytometry analysis on SN-MM cell lines. CD271 expression is reported on X axes; on y-axes side scatter (SSC) is reported. Grey dot plots are negative controls. Dot plots are representative of two independent experiments. **B** Representative images of immunohistochemistry analysis of parental tumor biopsies, stained as indicated. Green rectangles mark cells co-expressing ZEB1 and CD271 and lacking MITF and CDH1. Black arrows point cells co-expressing MITF and CDH1 and lacking ZEB1 and CD271. Magnification 400x, scale bar 25 µm

We subsequently tested the in vivo tumorigenic potential by subcutaneous injection of SN-MM cells in the flank of NOD/SCID mice (n = 6 mice/line). All SN-MM grew progressively and formed subcutaneous tumor nodules showing different tumorigenic potential (Additional file 6: Table S3). On histology, similarly to tumor cells of the parental biopsies, cell-derived xenograft (CDX) cells displayed a dominant epithelioid morphology (Fig. 4 and Additional file 6: Table S1) and a phenotype coherent with a melanocytic identity (Fig. 4 and Additional file 7: Figure S8). Moreover, as confirmed for SN-MM3 CDX cells, they also share the molecular driver NRAS p.Gly12Asp with the corresponding parental tumor and cell line (Additional file 7: Figure S9). In term of growth, F0 generation of SN-MM3 and SN-MM4 showed a lower percentage of tumor take, likely associated to their limited fitness (Additional file 7: Figure S3B–D); however, subcutaneous injection of SN-MM4 F1 resulted in a more rapid tumor growth compared to the F0 generation (n = 6, 12 *versus* 24 weeks; Additional file 6: Table S3). Cell-derived Xenografts (CDX) growth was followed up to 300 mm^3^ (8–24 weeks) and tumor plugs were harvested for microscopic analysis. CDX showed heterogeneity in terms of morphology and melanoma antigens expression (Fig. 4, Additional file 7: Figures S2 and S8). Of note, SN-MM1-CDX, SN-MM2-CDX, SN-MM3-CDX and SN-MM4-CDX contain clusters of small SOX10^+^ neoplastic cells and mostly lacking MITF, MART-1, HMB45, and tyrosinase expression. MITF^−/low^ melanoma cells in CM correspond to melanoma-initiating cells (MIC) [62]. We investigated the distinctive phenotype of these MITF^−/low^ melanoma cells unveiling the loss of E-cadherin (CDH1) accompanied by concomitant increased expression of N-cadherin (CDH2) (Fig. 5), a feature associated with epithelial-to-mesenchymal transition (EMT) in various cancers including melanoma [63]. Furthermore, MITF^−/low^ melanoma cells strongly expressed the Nerve Growth Factor Receptor (NGFR/CD271) identified as a marker of MICs [64] and, partially, the epithelial-to-mesenchymal transition (EMT) marker ZEB1 (Fig. 5). This population was detected also in the CDX obtained from SN-MM4 F1 generation (Additional file 7: Figure S10B). Based on a recent observation [65], this phenotype corresponds to MIC. Notably, SN-MM5-CDX maintained a high expression of nuclear MITF along with the absence of CDH1^−^/CDH2^+^/ZEB1^+^/CD271^+^ cells, thus suggesting a relevant role for MITF inhibition in sustaining the MIC reservoir in MM (Fig. 5). We also confirmed CD271 expression in SN-MM1-4 cell lines by flow cytometry (96.4% SN-MM1; 57.3% SN-MM2; 82.4% SN-MM3; 92.7% SN-MM4) and the negativity of SN-MM5 for CD271 (Fig. 6A). Analyzing the parental MM tumors, we could detect a variable fraction of MITF^−/low^/CDH1^−^/ZEB1^+^/CD271^+^ cells (Fig. 6B), proposing the existence of a MIC subpopulation in the primary tumor mass. However, it should be noted that only a fraction of CD271^+^ melanoma cells expressed a phenotype coherent with MIC, thus suggesting heterogeneity within this melanoma cell population. In addition to fully differentiated clones (MITF^+^/CD271^−^/ZEB1^−^/CDH1^+^; Fig. 6B and Additional file 7: Figure S10), we identified MITF^+^/CD271^+^/ZEB1^−^/CDH1^−^ cells showing an intermediate phenotype (Additional file 7: Figure S10A) along differentiation from MIC or EMT. CD271 is likewise expressed in neural crest stem cells, melanocytes, and melanoma cells regulating phenotype switching towards a stem-like or mesenchymal state [66].

### SN-MM cell lines are sensitive to cisplatin, but not to temozolomide

As reported in a phase II randomized trial, patients with MM in stage II/III treated with temozolomide plus cisplatin as systemic adjuvant therapy showed significant improvements in relapse-free and overall survival than those treated with either high-dose IFN-a2b or surgery alone [67]. Based on these premises, we explored the role of DNA alkylating agents in SN-MM. Cell lines were treated with increasing concentrations of cisplatin (0.625–20 µM) or temozolomide (3.125–200 µM) and then analysed by MTS assay. The dose–response curves revealed that cisplatin induced a decreased viability in all SN-MM cell lines, although with different sensitivity (Additional file 7: Figure S11A). Sigmoidal concentration–response function was applied to calculate the IC50 values of cisplatin, ranging from 2.4 µM to 9.7 µM among different cell lines (Additional file 6: Table S4). The cisplatin was highly active in inducing cytotoxicity in three out of five SN-MM, as its efficacy reached about 95% at the highest concentration tested (Additional file 6: Table S4). On the contrary, temozolomide treatment did not significantly affect SN-MM cells viability, as shown in dose–response curves (Additional file 7: Figure S11B and Additional file 6: Table S4). The temozolomide reached maximum efficacy in SN-MM5 that was about 35% at the highest concentration tested (Additional file 6: Table S4**).**

### Proteomic profile of SN-MM cell lines identifies cancer-associated pathways

**Fig. 7:**
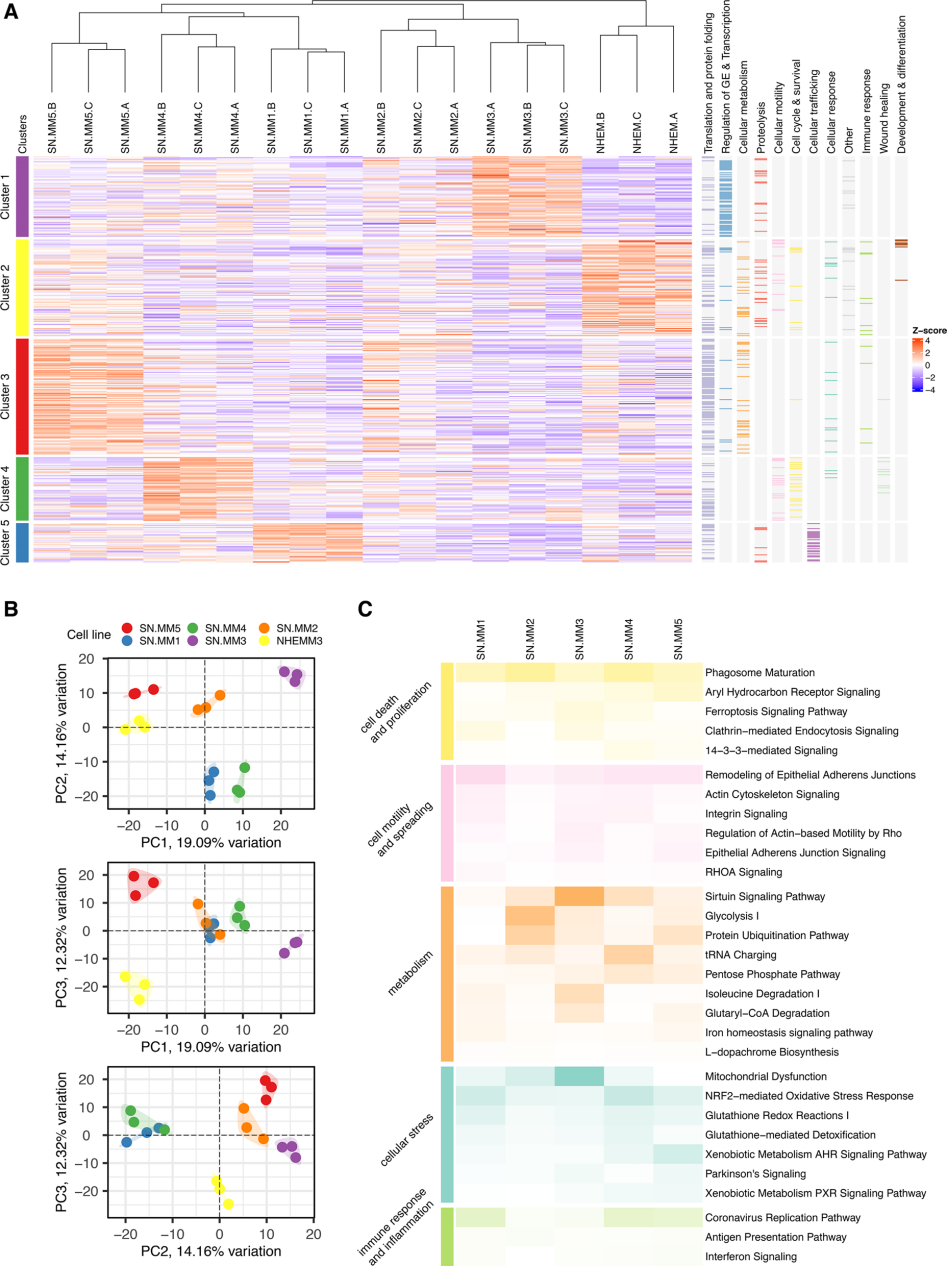
Proteomic profile of SN-MM cell lines. **A** Heatmap showing the expression of proteins involved in the significant up-regulated biological processes (393/1148 quantified, right panel); the five proteins’ clusters are highlighted and each of them is up-regulated in a different SN-MM cell line. **B** Scatter plots showing the combined projections of the first three components of a principal component analysis (PCA) run considering the whole protein expression on the cell lines’ samples. **C** Heatmap reporting the significant Canonical Pathways differentially regulated in all SN-MM cell lines versus normal melanocytes cell line (NHEM); color intensity is inversely proportional to p-values

We further characterized SN-MM cell lines by a mass spectrometry-based untargeted proteomic approach, which allowed the identification of 1148 measurable proteins in our cellular models. Unsupervised k-means clustering analysis identified five clusters of proteins selectively highly expressed in different samples (Additional file 7: Figure S12A-B and Additional file 2). Each protein cluster corresponds to an SN-MM sample, except for SN-MM2. By GO enrichment analysis, the five identified protein clusters showed to be enriched in various relevant biological processes (Fig. 7A and Additional file 3). By principal component analysis (PCA) projections of the same data set (Fig. 7B), SN-MM5 and NHEM resulted in proximity along PC1 projection, whereas normal melanocytes segregated from the remaining SN-MM cell lines made up the large part of the PC3 projection. The STRING functional enrichment analysis of cellular components confirmed that the melanotic SN-MM5 cell line has a greater number of up-regulated melanosome proteins than other amelanotic SN-MM cell lines (Additional file 7: Figure S12C-D and Additional file 6: Table S5), supporting a higher differentiated phenotype, also consistent with data from TEM and immunohistochemistry.

By GSEA, we performed a pathway enrichment analysis of the differentially expressed proteins in SN-MM cell lines compared to NHEM (Additional file 7: Figure S12E-F). Emerging cancer-associated pathways included the biosynthesis of nucleotides, peptides, and phospholipids and insulin signaling, along with down-regulation of the oxidative phosphorylation and monocarboxylic acid biosynthesis (Additional files 4 and 5), consistent with a cancer cell profile, as also indicated by a recent pan-cancer proteomic analysis [68]. Moreover, SN-MM cell lines are commonly defined by a dynamic chromatin remodeling, alternative splicing, processing and transport of mRNA (Additional file 5).

To identify relevant Canonical Pathways and Upstream Regulators of SN-MM cell lines we used IPA, corroborating the involvement of cancer-associated pathways in SN-MM cell lines compared to NHEM (p < 0.05) (Fig. 7C). Among relevant pathways, ferroptosis, a programmed cell death induced by excessive iron-dependent lipid peroxidation, is frequently suppressed in tumors, and likely in SN-MM (Fig. 7C). In addition, Mitochondrial Dysfunction, NRF2-mediated Oxidative Stress Response, Glutathione Redox Reactions and Glutathione-mediated Detoxification Canonical Pathways supported the dysregulation of both oxidative stress and ferroptosis in SN-MM cell lines (Fig. 7C). Of relevance, we found Glycolysis and Pentose Phosphate Pathway (PPP) that are important in cancer cell metabolism and survival (Fig. 7C); aerobic glycolysis sustains a high proliferative rate of tumor cells under nutrient starvation, while the PPP plays a crucial role in combat oxidative stress. Sirtuin signaling also plays a critical role in metabolic reprogramming in tumor cells. These data provide a novel insight into the metabolic landscape sustaining MM evolution. Finally, 14-3-3-mediated signaling initiates the mitogenic response of cells to growth factors and is involved in the process of tumorigenesis. Aryl hydrocarbon receptor (AHR) and xenobiotic metabolism signaling mediated most of the responses to carcinogen exposure; moreover, AHR interacts with signaling pathways involved in cell cycle progression, cell proliferation, apoptosis and tumorigenesis.

IPA upstream regulator analysis identifies functionally relevant upstream molecules (Additional file 7: Figure S13). Particularly, the transcription factors MITF and TFEB, belonging to a subfamily of related basic helix-loop-helix-leucine zipper proteins, are significantly inhibited upstream regulators (Additional file 7: Figure S13 and S14A-D). The onco-suppressor TP53 and the oncogenic transcription regulator MYC are respectively inhibited and activated upstream regulators in SN-MM5 (Additional file 7: Figure S13). Moreover, serine/threonine kinase STK11 and the transcription regulator NFE2L2 are among the inhibited upstream regulators, whereas tyrosine phosphatase PTP4A1 and sirtuin SIRT1 are predicted as activated upstream regulators (Additional file 7: Figure S13). STK11 is a tumor suppressor involved in cell motility and metabolism and NFE2L2 plays a key role in oxidative stress response, while PTP4A1 has been recently identified as a novel therapeutic target in cancer.

Therefore, the proteomic landscape of SN-MM cell lines indicates a cancer cell profile defined by a less differentiated and more invasive phenotype combined with metabolic hallmarks of cancer and sustained cell proliferation.

### Functional activation of PI3K-Akt-mTOR pathway in SN-MM.

**Fig. 8:**
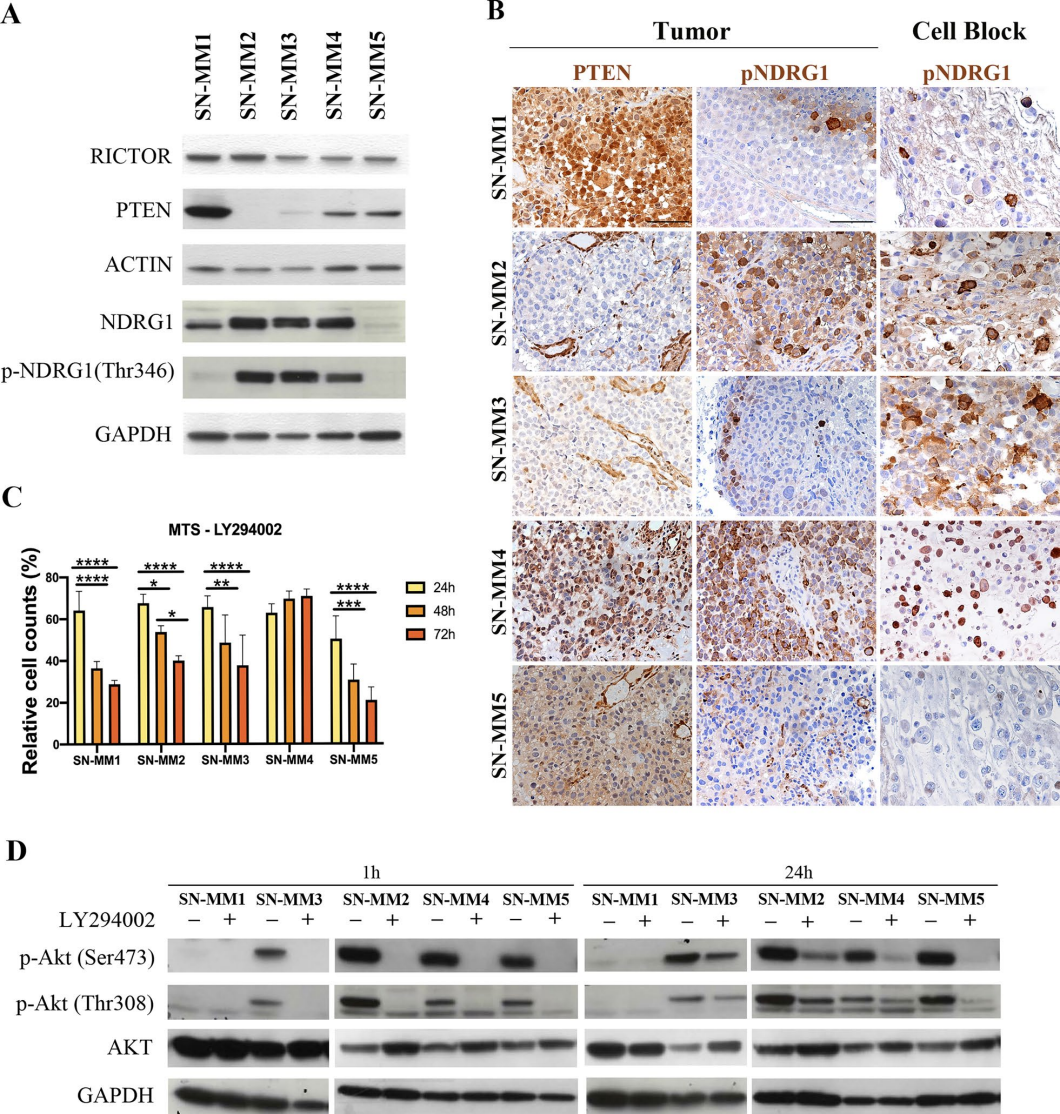
PI3K/Akt/mTOR pathway activation and PTEN loss in SN-MM. **A** Western blots showing RICTOR, PTEN, NDRG1 and pNDRG1(Thr346) expression in SN-MM cell lines cultured in complete RPMI medium; ACTIN and GAPDH are used as housekeeping control. **B** Immunohistochemistry for PTEN and pNDRG1(Thr346) on parental tumor biopsies (left panels) and pNDRG1(Thr346) on cell blocks (right panels) from SN-MM samples. Lack of PTEN expression is shown in SN-MM2 and SN-MM3 parental tumors; a significant PTEN reduction is observed also in SN-MM5. pNDRG1 is strongly expressed in SN-MM2, SN-MM3 and SN-MM4 cell lines, and correspondingly in SN-MM2 and SN-MM4 parental tumors. Sections are counterstained with haematoxylin. Magnification 200X; scale bar 100 µm. **C** Viability of SN-MM cell lines treated with the PI3K inhibitor measured by MTS assay. Histograms represent the percentage of viable cells after administration of LY294002 (50 µM) for 24 h, 48 h, and 72 h, relative to untreated SN-MM cells (n = 3–5). Two-way ANOVA statistical analysis and Bonferroni’s multiple comparison post-test have been performed. *p < 0.05; **p < 0.01; ***p < 0.001; ****p < 0.0001. **D** Western blots showing Akt expression and its phosporylation p-Akt(Ser473) and p-Akt(Thr308) in SN-MM cell lines cultured in complete RPMI medium ± LY294002 (50 µM) for 1 h and 24 h. GAPDH is used as housekeeping control

As previously reported by our group, SN-MM are characterized by recurrent expression of phosphorylated Akt on melanoma cells from clinical samples, suggesting a relevant role for the activation of the PI3K-Akt-mTOR pathway in these neoplasms [16]. In our SN-MM cohort, PI3K-Akt-mTOR pathway activation was also observed in MM cases lacking *PIK3CA* mutations and showing PTEN expression, indicating a complex genetic and epigenetic basis for this finding [16, 69]. Based on the proteomic analysis, we found that RICTOR, a subunit of mTORC2 complex, is the most significantly activated upstream regulator in SN-MM cell lines (Additional file 7: Figure S13 and S14E-H). Accordingly, we could detect strong RICTOR expression in all SN-MM cell lines by western blot (Fig. 8A). Moreover, we observed a strong signal for phosphorylation of N-Myc downstream-regulated gene 1 (NDRG1), a distal effector of mTORC2 activation [70], in SN-MM2, SN-MM3 and SN-MM4 lines (Fig. 8A, B) as well as on SN-MM2 and SN-MM4 corresponding parental tumor biopsies (Fig. 8B), suggesting positive selection of pNDRG1^+^ tumor cells in vitro. These data are further supported by the gain of 5p15 and 8q24 loci, including *RICTOR* and *NDRG1* genes, respectively (Fig. 3A, B). Based on these premises, we explored PI3K-Akt-mTOR activation in SN-MM measuring pAktSer473 and pAktThr308 protein expression by western blot. pAktSer473 and pAktThr308 were expressed in all SN-MM lines and resulted constitutive in SN-MM2, SN-MM3 and SN-MM5 (Additional file 7: Figure S15A). NDRG1 is phosphorylated by SGK1, a direct substrate of mTORC2, while pAktSer473 is regulated by mTORC2 and promotes cell growth and survival [70, 71]; therefore, our results reveal mTORC2 activation in SN-MM cell lines. Moreover, Akt is activated by PI3K-mediated phosphorylation at the Thr308 site. We subsequently tested PTEN expression and found protein loss in SN-MM2 and SN-MM3 (Fig. 8A); PTEN expression was entirely consistent between SN-MM cell lines and parental tumor biopsies (Fig. 8B). In SN-MM5, PTEN expression resulted diminished in the parental tumor, thus explaining the Akt constitutive activation (Fig. 8B and Additional file 7: Figure S15A) [72, 73]. PTEN loss is supported by gene deletion (Fig. 3B) or loss-of-function mutation [74]. These findings confirm that PI3K-Akt-mTOR activation in MM is partially sustained by PTEN loss.

To address the role of the PI3K pathway in SN-MM cell survival, cell lines were treated with increasing doses of the PI3K chemical inhibitor (LY294002). LY294002 treatment reduced SN-MM cell viability in a dose-time-dependent manner (Additional file 7: Figure S15B-E); in particular, the concentration of 50 μM significantly decreased the number of viable cells over time in all but one SN-MM cell lines (Fig. 8C). As expected, LY294002 induced a significant inhibition of AKT phosphorylation at both activation sites (Ser473 and Thr308) after 1 h of treatment (Fig. 8D). However, AKT phosphorylation was partially rescued after 24 h of treatment with LY294002, suggesting the existence of a feedback mechanism downstream of PI3K, including mTORC2 activation, or selection of intrinsic resistant subclones that could restore AKT activation (Fig. 8D). These findings are in keeping with the relevant role of the activated PI3K-Akt-mTOR pathway in MM; loss of PTEN together with still unknown genomic aberrations might account for this observation.

## Discussion

Compared to the cutaneous counterpart, MM has been poorly investigated due to its rarity and lack of pre-clinical models. Generation of MM cell lines from the primary mucosal site is challenging with very little published. Moreover, none of the reported cell lines originated from MM of the sinonasal cavity [75–77]. The first well-validated MM cell line, generated from an oral cavity MM, has been very recently proposed [78]. This MM line shows low tumor-mutational burden, a lack of known oncogenic drivers, and is chemo-resistant to the vast majority of compounds currently approved for the melanoma treatment (BRAFi/MEKi, C-KIT inhibitors, anti-angiogenic kinase inhibitors, DNA-alkylating agents) [78]. Very recently, a set of organoids have been established from human oral MM suggesting NGFR as relevant biomarker for resistance to anti–PD-1 therapy [79]. In the present study, we could generate five novel human SN-MM cell lines (success rate over 10%) and confirm their identity through cell morphology, ultrastructural features, cell phenotype, and proteomic profile. SN-MM cell lines were tumorigenic and contain clusters of poorly differentiated MICs, resembling parental SN-MM tumors. Of clinical relevance, they resulted sensitive to cisplatin, but not to temozolomide, at variance with the proposed treatment protocol [67, 80]. The introduction of immunotherapies has led to a shift in the systemic treatment of MM, in the adjuvant setting and for advanced disease. Their proteomic profile is consistent with transformed melanocytes showing a heterogeneous degree of melanocytic differentiation along with activation of cancer-related genes and pathways. By chemical inhibition, we could demonstrate a functional role for PI3K/Akt/mTOR to sustain tumor cell viability. These cellular systems might represent unique preclinical tools for a better understanding of the molecular landscape and the immunobiology of these neoplasms (manuscript in preparation) and, as an extension, to MM from other sites. These studies, will likely results in innovative treatment options and corresponding tissue biomarkers. Although with different levels of differentiation [81], SN-MM are *bona fide* melanocytic, as demonstrated by their expression of conventional melanocytic markers, largely comparable to the parental tumor. SOX10 and PRAME resulted positive in all tumor cells, whereas the other melanocytic markers showed extreme heterogeneity. It should be noted that phenotypic plasticity and undifferentiation (i.e. loss of diagnostic immunomarkers) within primary and metastatic malignant melanomas is an uncommon but well-documented phenomenon [82, 83]. TEM analysis unveiled that SN-MM cells are endowed with melanosomes at different stages of development. Specifically, SN-MM5 is enriched in mature and immature melanosomes, whereas the melanosomes are rare or mainly immature in the remaining SN-MM cell lines. Accordingly, only SN-MM5 is melanotic in vitro and maintains strong and diffuse expression of HMB-45 and tyrosinase [50]. In line with these data, one-third of head&neck-MM appear as amelanotic [49]. It should be noted that TEM landmark studies confirmed various degrees of differentiation also in primary CM [84].

How this level of de-differentiation account for the biological features of human MM is unknown. The malignant transformation of skin melanocytes occurs through a well-established sequential accumulation of genetic and molecular alterations involving precursors [7, 85]. On the contrary, limited data are available on the cellular and molecular features sustaining mucosal melanocyte transformation and MM progression [86].

As revealed by aCGH, structural rearrangements on SN-MM cell lines targeted a wide number of chromosome regions, including 1q, 5p, 6p, 6q, 8q and 9p, with a common consequence being gain of oncogenes, such as *TERT*, *MYC*, *KRAS*, or loss of tumor suppressor genes, such as *ARID1B* and *CDKN2A*. The loss of p16^INK4a^ protein expression further support these data, suggesting that MM might benefit from therapeutic strategies targeting *CDKN2A* loss, including the pharmacological inhibition of CDK4/6 (i.e. palbociclib and ribociclib) that are targets of p16 ^INK4a^ [87]. Copy number gain of *BRAF* and copy number deletion of *NF1* were also observed in most SN-MM cell lines, suggesting potential drivers for these melanocytic tumors. Moreover, copy number alterations involving components of the PI3K/Akt/mTOR pathway were found in SN-MM cell lines, including recurrent gains of *AKT*, *RICTOR*, *RPTOR*, *NDRG1*, *RPS6KB1*, *RHEB*, and loss of *SGK1*, supporting a relevant role for PI3K/Akt/mTOR pathway in controlling cell growth in these tumors. Based on the proteomic analysis performed in this study, several pathways are involved in the SN-MM cells maintenance, including those controlling cell cycle and proliferation, senescence, cell motility, and metastatic spreading, the epigenetic and transcriptional regulation of gene expression, cancer cell metabolism and cellular response to oxidative stress as well as immune response [10, 88].

SN-MM cell lines demonstrated different tumorigenic properties in vivo, that could be partially explained by their intrinsic fitness and differentiation trajectories, including a stem-like quiescent state, likely regulated by the surrounding microenvironment and marked by MITF expression [89]. Invasive MITF^−^ cells require a longer period to initiate in vivo growing tumor by comparison with MITF^+^ proliferative cells [89]. Of note, based on the microscopic analysis of mouse skin xenografts of SN-MM cells, we could identify a proliferation of MITF^low^/CDH1^−^/CDH2^+^/ZEB1^+^/CD271^+^ melanoma cell clusters losing most melanocytic markers, thus suggesting a MIC identity, as proposed in CM [64, 90–92]. Although this outcome requires a set of additional experiments to be completely understood, this is in keeping with the existence of a dedifferentiated MM component endowed with an increased aggressiveness [93, 94] and tumor-initiating cell features [34, 95]. The malignant transformation of melanocytes involves the EMT that convey invasiveness and tumor-initiating potential. The “cadherin-switch” is critical for malignant melanocytes to escape from the primary tumor mass [96]; cell–cell and cell–extracellular matrix adherence junctions are remodeled leading to cancer cell dissemination, and a new transcriptional program is activated. Due to epigenetic plasticity, the EMT is a reversible process and can give rise to cancer stem cells during the metastatic dissemination.

SN-MM lines display a partial melanocytic differentiation also loosing MITF; moreover, clusters of MITF^low^/CDH1^−^/CDH2^+^/ZEB1^+^/CD271^+^ cells have been identified in mouse xenografts and parental biopsies of SN-MM. This population is likely driven by their MITF^low^ phenotype in their MIC functions and might undergo transition to differentiated melanoma cells. The upstream regulator analysis revealed inhibition of the MiT/TFE family of transcription factors, including MITF, TFEB, and TFE3, representing critical hubs in melanocytes differentiation, melanosome biogenesis, melanin biosynthesis, and melanosome transport [97]. MITF coordinates many biological properties of melanocytes and might be used as a key molecular marker to distinguish between differentiated, proliferative (MITF^+/high^), or invasive (MITF^−/low^) melanoma phenotypic states [98]. Specifically, MITF^high^ promotes melanocyte differentiation, proliferation, and survival, whereas MITF^low^ leads to invasion and senescence [95, 97]. Moreover, MITF can regulate DNA damage repair, telomere maintenance [99], and cell metabolism as mitochondrial biogenesis and oxidative phosphorylation [100], activates SIRT1 expression, and together with TFEB controls lysosome biogenesis and autophagy [95, 97, 101]; therefore, the MITF^low^ phenotype might justify some of the enriched pathways of proteomic analysis.

Previous data from our group [16] and results from this study suggest a relevant role for the activation of the PI3K-Akt-mTOR pathway in MM. Activation might rely on PTEN loss in at least one-third of MM [16]. Accordingly, co-existing PTEN loss and constitutive Akt activation were observed in two out of five cell lines. Novel treatment options have been recently proposed for PTEN-deficient cancer [102]. PTEN loss sustain stem cell self-renewal and has been associated with cadherin switch [103] during CM progression. The cadherin switch is regulated via PI3K/Akt by transcriptional up-regulation of Twist and Snail transcription factors [103]. The observed outcome of PI3K-Akt-mTOR inhibition on SN-MM cells viability might thus rely on a direct effect also on the MIC reservoir. By flow cytometry analysis, we found that the NGFR/CD271^+^ cells correspond to a significant fraction of the SN-MM population; however, this population contain also cells lacking a MIC phenotype. The MIC are the major contributors to tumor dissemination and persistence and tightly linked to drug resistance. Therefore, effort to identify and characterize MIC within the CD271^+^ population are mandatory to identify appropriate therapeutic targets for this neoplasm [104]. Furthermore, it should be noted that CD271/NGFR has been emerged as a relevant factor in the resistance to anti–PD-1 therapy in oral MMs, paving the way for new combination therapies with anti-PD-1 [79]. The combination of PI3Ki with selective blocking of mTORC2 activity, by knocking-down RICTOR [105], could avoid feedback loops and overcome chemo-resistance mechanism.

## Conclusions

The cellular systems generated in this study recapitulate primary MM of the sinonasal tract. Microscopic analysis of SN-MM derived xenografts and patient’s tumor samples identified clusters of melanoma-initiating cells. In addition, we suggested a functional role for the PI3K-Akt-mTOR pathway in these neoplasms with potential clinical relevance. These novel and unique cellular systems might offer relevant tools to further expand our understanding of the immunobiology of these rare neoplasms and identify innovative treatment options, with patient selection based on biomarkers validation on human samples.

### Supplementary Information

**Additional file 1. **Pigmentation of SN-MM cell pellets.

**Additional file 2. **List of proteins belonging to clusters obtained from unsupervised k-means clustering analysis.

**Additional file 3. **List of enriched GO biological processes (FDR < 0.05) describing the five clusters of selectively highly expressed proteins identified by mass spectrometry.

**Additional file 4 **List of significantly enriched pathways (ES > 0.25; ES < − 0.25) in each SN-MM cell line versus NHEM from GSEA.

**Additional file 5. **List of significant pathways commonly enriched (ES > 0.25; ES < − 0.25) in five SN-MM cell line versus NHEM from GSEA.

**Additional file 6: Table S1. **Demographic and clinico-pathological characteristics of the SN-MM patients. **Table S2. **List of primary antibodies used for immunohistochemistry and immunoblotting. **Table S3.** In vivo growth kinetics of SN-MM Cell-derived Xenografts. **Table S4. **Efficacy of chemotherapeutic treatment in SN-MM cell lines. **Table S5.** List of proteins of the melanosome cellular component from STRING functional enrichment analysis up- and down-regulated in SN-MM5 compared to other cell lines.

**Additional file 7: Figure S1. **IHC analysis of SN-MM parental tumor biopsies and corresponding cell blocks (CB). Expression of melanocytic markers MITF, HMB45, MART, TYROSINASE, S100 and tumor associated fibroblast marker α-SMA. Sections are counterstained with hematoxylin. Parental tumors: Magnification 200X, scale bar 100 µm. CB: Magnification 400X, scale bar 50 µm. **Figure S2. **Scoring of melanocytic biomarkers in patient’s tumors, their corresponding SN-MM cell lines and cell-derived xenografts. Heatmap summarizing immunohistochemical expression of melanocytic biomarkers and α-SMA on the SN-MM cell lines cell block, patient’s tumor biopsies and mouse skin xenografts. Four-tiered scoring system was adopted, as described in methods section. NA: not assessed. **Figure S3. **Proliferation index and density of mitotic cells in SN-MM cell lines. **A **Representative images of ki67 and ph-H3 staining in SN-MM5 cell-block. Sections are counterstained with hematoxylin. Magnification 400X, scale bar 50 µm. **B**, **C** Quantification of ki67 and ph-H3 staining in SN-MM cell blocks. Histograms represent the percentage of positive cells out of total cells in three 20X field analyzed on digitalized slides. **D** Proliferation of SN-MM cell lines measured by MTS assay at 24 h, 48 h, and 72 h (n = 5). Histograms represent the absorbance at 490 nm that is directly proportional to the number of live cells in culture. One-way ANOVA statistical analysis and Bonferroni’s multiple comparison post-test have been performed. *p < 0.05; ** p < 0.01; *** p < 0.001. **Figure S4.** Immunofluorescence staining of melanocytic biomarkers in SN-MM cell lines. SN-MM cells were cultured and stained as labeled. Cell nuclei were stained with DAPI (blue). Representative images of expression of MART (green), SOX10 (red) and S100 (green) are shown. Scale bar 20 µm. **Figure S5. **Ultrastructural features of SN-MM cell lines. TEM images showing cell morphology and heterogeneity among SN-MM cell lines (**A**–**F**); electron dense melanocytes are more evident in NHEM (**F**) and SN-MM5 (**E**) cell lines. Scale bars are 10 µm. **Figure S6. **Ultrastructural analysis of melanosomes on SN-MM cell lines. TEM images showing low electron dense pre-melanosomes in SN-MM1 (**A**), SN-MM2 (**B**) and SN-MM3 (**E**), early stage melanosomes presenting a fibrillar pattern in SN-MM2 (**C**, **D**), SN-MM3 (**F**, **G**), and SN-MM5 (**I**), and high electron dense mature melanosomes in SN-MM4 (**H**) and SN-MM5 (**I**). Scale bars are 1 µm (**A**, **B**, **H**, **I**), 500 nm (**C**, **E**, **G**) and 200 nm (**D**, **F**). **Figure S7.** Gains and losses of chromosome regions in five SN-MM cell lines. Overview of rearranged chromosomal regions in SN-MM1 (**A**), SN-MM2 (**B**), SN-MM3 (**C**), SN-MM4 (**D**), SN-MM5 (**E**), and SN-MM5.P1 (**F**) cell lines as detected by array-comparative genomic hybridization (aCGH). Red and green lines on the side of each chromosome represent losses and gains, respectively. **Figure S8. **IHC analysis of SN-MM parental tumor biopsies and corresponding cell-derived xenografts. Expression of melanocytic markers SOX10, MITF, MART, TYROSINASE, HMB45, and S100. Sections are counterstained with hematoxylin. Magnification 200X, scale bar 100 µm. **Figure S9. **NRAS mutation in SN-MM3. Sequence chromatograph of NRAS exon 2 of genomic DNA from SN-MM3 cell line (upper panel), parental tumor biopsies (middle panel), and cell-derived xenograft (lower panel) showing the c.35G>A mutation resulting in NRAS p.Gly12Asp. Reverse sequence is shown. **Figure S10.** MIC and intermediate phenotypes in parental tumors and SN-MM-CDX from F1 generation. Sections are from parental tumor biopsies (**A**) and CDX obtained from SN-MM4 F1 generation (**B**), stained as indicated. Yellow rectangles mark cells co-expressing MITF and CD271 and lacking ZEB1 and CDH1. Green rectangles mark cells co-expressing ZEB1 and CD271 and lacking MITF and CDH1. Black arrows point cells co-expressing MITF and CDH1 and lacking ZEB1 and CD271. Magnification 40x, Scale bar 25 µm. **Figure S11. **Dose-response curves of cisplatin and temozolomide in SN-MM cell lines. SN-MM cells were treated with cisplatin (0.625–20 µM; **A**) for 72 h (SN-MM1; SN-MM2; SN-MM3; SN-MM5) or 120 h (SN-MM4) or with temozolomide (3.125–200 µM; **B**) for 72 h. Results are expressed as percentages of viable cells *versus* untreated cells ± SD of three independent experiments, run in triplicate. Two-way ANOVA statistical analysis and Bonferroni’s multiple comparison post-test have been performed. For concentrations starting from 20 µM to 0.625 µM vs. untreated cells (**A**); *p < 0.05; ** p < 0.01; *** p < 0.001. **Figure S12. **Differentially expressed proteins and pathways among SN-MM cell lines and NHEM.** A** Plot showing the optimal number of five clusters of quantified proteins, for the k-means clustering, maximizing the average silhouette width. **B** Heatmap showing the scaled protein expression; unsupervised k-means clustering of proteins is applied highlighting groups of proteins with selective upregulation in five groups of samples, corresponding to different specific cell lines. **C**, **D** Histograms showing the numbers of up- and down-regulated melanosome’s proteins (**C**) and the ratios between up- and down-regulated proteins (**D**) in SN-MM5 compared to NHEM and other SN-MM cell lines, obtained from the STRING functional enrichment analysis. **E**, **F** Venn diagrams depict the number of positively and negatively enriched pathways among SN-MM cell lines. The number of positively (ES > 0.25; **E**) and negatively (ES < − 0.25; **F**) enriched pathways (p-val < 0.05) in each SN-MM cell line compared to NHEM are reported in the histograms. The five-group Venn diagrams showed 15 up-regulated (ES > 0.25; **E**) and 3 down-regulated (ES < − 0.25; **F**) commonly enriched pathways in all SN-MM cell lines. Venn diagrams are realized by using jvenn at the following linkhttp://jvenn.toulouse.inra.fr/app/example.html. **Figure S13. **Upstream Regulators in the SN-MM cell lines. Heatmap showing the predicted expression of upstream regulators (z-score) by IPA in the SN-MM cell lines compared to NHEM. Upstream regulators with a significant (p < 0.05) and biological relevant activation (Z-score ≧ 2) or inhibition (Z-score ≦ -2) at least in one sample are shown. Significant and biological relevant upstream regulators are highlighted with labels showing the value of the Z-score. Not available data for an upstream regulator are indicated in light green color. **Figure S14. **Networks of upstream regulators in SN-MM cell lines. **A**–**D** The IPA upstream regulator MITF is significantly inhibited (z-score ≤ − 2) in four out of five SN-MM cell lines. **E**–**H** The IPA upstream regulator RICTOR is significantly activated (z-score ≥ +2) in four out of five SN-MM cell lines. **Figure S15**. Activation of Akt and sensitivity to PI3K inhibition in SN-MM cell lines. **A** Western blots showing Akt and phosporylated-Akt(Ser473, pAkt) and Akt(Thr308, pAkt) expression in SN-MM cell lines cultured in complete RPMI medium (Ctrl), after o/n starvation (1% FBS; Starv), and replenished with 10% FBS for 30 minutes (FBS). GAPDH is used as housekeeping control. **B**–**D** Dose-response curves of SN-MM cell lines treated with LY294002 chemical inhibitor (1 µM, 5 µM, 10 µM, and 100 µM) after 24 h (**B**), 48 h (**C**) and 72 h (**D**) (n = 5 for SN-MM1, SN-MM5; n = 4 for SN-MM2, SN-MM3; n = 3 for SN-MM4). Half maximal effective concentration (EC50) of LY294002 is reported for each SN-MM cell line at 24 h, 48 h and 72 h (**E**).

## Data Availability

All data generated or analyzed during this study are included in this published article and its supplementary information files. The mass spectrometry proteomics data generated and analyzed during the current study are available in the ProteomeXchange Consortium via the PRIDE partner repository with the dataset identifier PXD037551.

## Abbreviations

MM: Mucosal melanoma
CM: Cutaneous melanoma
BRAFi: BRAF inhibitors
ICI: Immune checkpoint inhibitors
TMB: Tumor mutational burden
SN-MM: Sinonasal mucosal melanoma
NHEM: Normal human epidermal melanocytes
IHC: Immunohistochemistry
H&E: Haematoxylin–eosin
TEM: Transmission electron microscopy
ER: Endoplasmic reticulum
CDX: Cell-derived xenografts
MIC: Melanoma initiating cells
EMT: Epithelial-to-mesenchymal transition
PCA: Principal component analysis
IPA: Ingenuity pathway analysis
