# Supplementary figures and images for: Novel cellular systems unveil mucosal melanoma initiating cells and a role for PI3K/Akt/mTOR pathway in mucosal melanoma fitness
Source: J Transl Med. 2024 Jan 8;22:35. doi: 10.1186/s12967-023-04784-2 (PMC10775657; doi:10.1186/s12967-023-04784-2)

## Slide 1
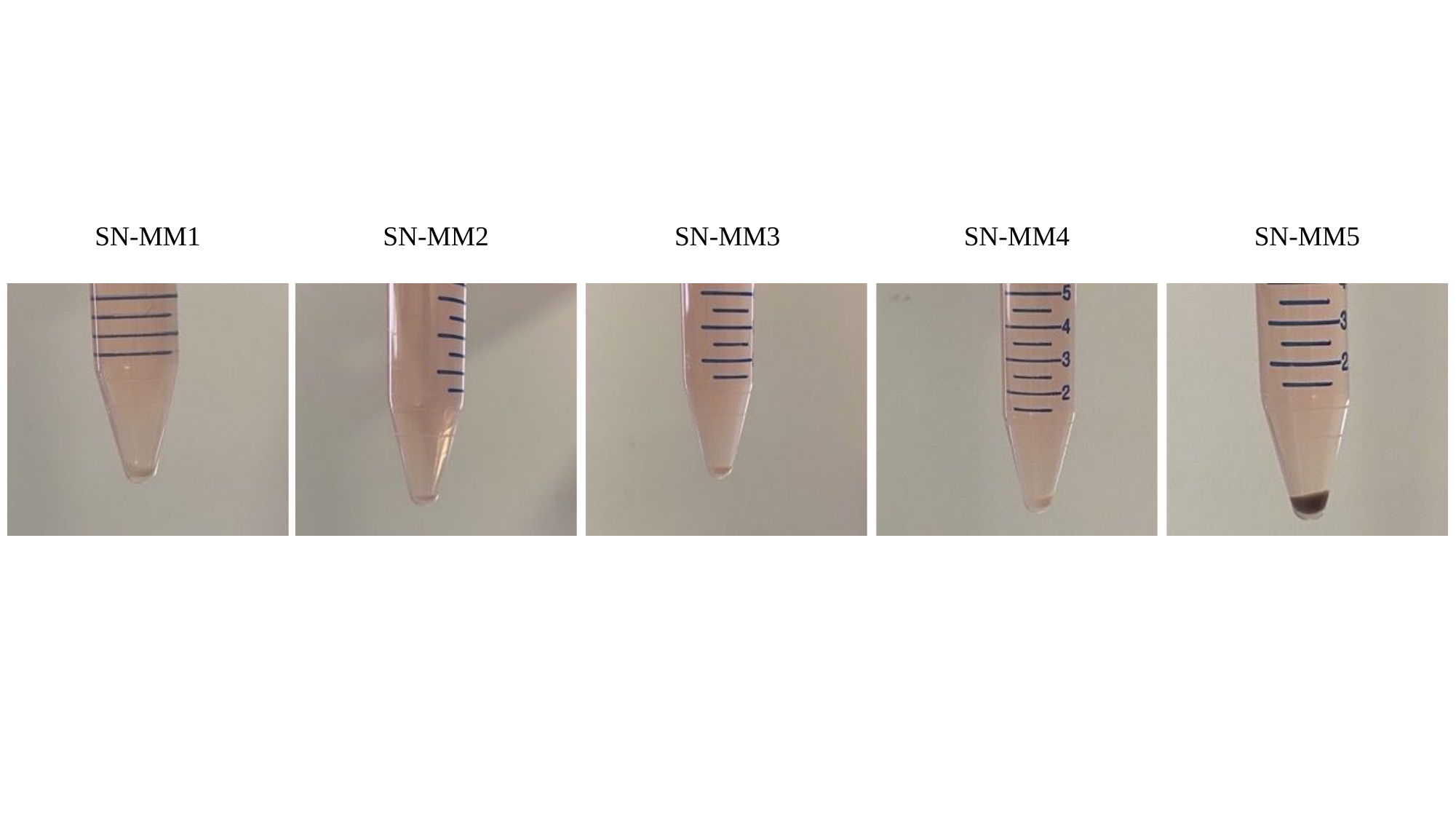

SN-MM1
SN-MM2
SN-MM3
SN-MM4
SN-MM5

Supplement: Supplementary file 1 — Additional file 1. Pigmentation of SN-MM cell pellets. [file 12967_2023_4784_MOESM1_ESM.pptx]
