# Supplementary material for: Novel cellular systems unveil mucosal melanoma initiating cells and a role for PI3K/Akt/mTOR pathway in mucosal melanoma fitness
Source: J Transl Med. 2024 Jan 8;22:35. doi: 10.1186/s12967-023-04784-2 (PMC10775657; doi:10.1186/s12967-023-04784-2)
Supplement: Supplementary file 6 — Additional file 6: Table S1. Demographic and clinico-pathological characteristics of the SN-MM patients. Table S2. List of primary antibodies used for immunohistochemistry and immunoblotting. Table S3. In vivo growth kinetics of SN-MM Cell-derived Xenografts. Table S4. Efficacy of chemotherapeutic treatment in SN-MM cell lines. Table S5. List of proteins of the melanosome cellular component from STRING functional enrichment analysis up- and down-regulated in SN-MM5 compared to other cell lines. [file 12967_2023_4784_MOESM6_ESM.docx]

Supplementary Tables

**Supplementary Table S1. Demographic and clinico-pathological characteristics of the SN-MM patients.**

| **SN-MM Patient** | **Gender** | **Age** | **Subsite** | **T** | **N** | **M** | **Morphological features** | **Treatment** | **Site of Recurrence** | **Follow-up (months)** | **Status** |
| --- | --- | --- | --- | --- | --- | --- | --- | --- | --- | --- | --- |
| #1 | F | 63 | Nasal cavity | 4b | 0 | 0 | Epithelial-like | EER and postoperative IMRT | T and N relapse | 5 | DOD |
| #2 | F | 68 | Nasal cavity | 4a | 0 | 0 | Epithelial-like | Endoscopic resection | Lung, brain, bone metastases | 13 | DOD |
| #3 | F | 86 | Nasal cavity | 4a | 0 | 0 | Epithelial-like | EER and postoperative IMRT | Distant Metastases | 29 | DOD |
| #4 | M | 43 | Nasal cavity | 3 | 0 | 0 | Epithelial-like | EER and postoperative carbon ion  radiotherapy | T and M relapse | 8 | DOD |
| #5 | F | 81 | Nasal Cavity | 3 | 0 | 0 | Epithelial-like | EER and postoperative IMRT | T and M relapse | 22 | DOD |

Abbreviations: EER, endoscopic endonasal resection; DOD, died of disease; IMRT, Intensity Modulated Radiation Therapy.

**Supplementary Table S2. Primary antibodies used in immunohistochemistry, immunofluorescence and immunoblotting**.

| **Antibody** | **Clone** | **Species** | **Dilution** | **Company** | **Cat.n.** |
| --- | --- | --- | --- | --- | --- |
| α-SMA | 1A4 | Mouse IgG2A | 1:200 | Thermo Fisher Scientific | MS-113-P0 |
| MITF | D5 | Mouse Ig1 | 1:50 | Dako | M3621 |
| MART-1 | A103 | Mouse IgG1K | 1:50 | Dako | M7196 |
| HMB-45 | HMB45 | Mouse IgG1K | 1:50 | Dako | M0634 |
| SOX10 | A-2 | A-2 Mouse IgG1K | 1:1500 | Santa Cruz Biotec | sc-365692 |
| S100 | Polyclonal | Rabbit | 1:300 | Dako | Z 0311 |
| Tyrosinase | T311 | Mouse IgG2A | 1:50 | Thermo Fisher Scientific | MS-800-P0 |
| PRAME | EPR20330 | Rabbit | 1:100 | Abcam | ab 219650 |
| Ki-67 | 30-9 | Rabbit | 1:1 | Roche | 790-4286 |
| p16INK4a | E6H4 | Mouse | 1:4 | CINtec | 06680003001 |
| phospho-Histone H3 | Polyclonal | Rabbit | 1:5000 | Epitomics | ab5176 |
| E-Cadherin | 4A2C7 | Mouse IgG1K | 1:30 | Thermo Fisher Scientific | 18-0223 |
| CDD2/N-Cadherin | Polyclonal | Rabbit | 1:70 | LSBio | LS-210521 |
| P75 NGF Receptor/ CD271 | EP1039Y | Rabbit | 1:400 | Abcam | ab52987 |
| ZEB1 | OTI3G6 | Mouse IgG2A | 1:6000 | Abcam | ab180905 |
| RICTOR | 1G11 | Mouse IgG | 1:1000 | Santa Cruz, Biotec. | sc-81538 |
| NDRG1 | D8G9 | Rabbit IgG | 1:1000 | Cell Signalling | 9485 |
| pNDRG1 (Thr346) | D98G11 | Rabbit IgG | 1:1000 | Cell Signalling | 5482 |
| AKT | C67E7 | Rabbit IgG | 1:1000 | Cell Signalling | 4691 |
| pAKT (Ser473) | D9E | Rabbit IgG | 1:2000 | Cell Signalling | 4060 |
| pAKT (Thr308) | 244F9 | Rabbit IgG | 1:1000 | Cell Signalling | 4056 |
| PTEN | 6H2.1 | Mouse IgG | 1:1000 | Dako | M362729-2 |
| GAPDH | D16H11 | Rabbit IgG | 1:1000 | Cell Signalling | 5174 |
| α-ACTIN | Polyclonal | Rabbit IgG | 1:1000 | Millipore-Sigma-Aldrich | A5060 |

**Supplementary Table S3. *In vivo* growth kinetics of SN-MM Cell-derived Xenografts.**

| **Cell line** | **Tumor take**  **(n° grown/n° injected)** | **Days**  **after tumor injection** | **Tumor volume mm^3^ (mean±SEM)** |
| --- | --- | --- | --- |
| SN-MM1 | 6/6 | 62 | 260 ± 20 |
| SN-MM2 | 4/4 | 47 | 301 ± 56 |
| SN-MM3 | 1/6 | 151 | 147 |
| SN-MM4 | 2/6 | 168 | 154 ± 20 |
| SN-MM5 | 6/6 | 38 | 228 ± 21 |
| SN-MM4.F1 | 6/6 | 84 | 286 ± 6 |

**Supplementary Table S4: Efficacy of chemotherapeutic treatment in SN-MM cell lines.**

| **CHT** | **Sensitivity and Efficacy** | **SN-MM1** | **SN-MM2** | **SN-MM3** | **SN-MM4** | **SN-MM5** |
| --- | --- | --- | --- | --- | --- | --- |
| Cisplatin | IC50 µM (95% CI) | 4.21  (3.35-5.29) | 8.13  (6.65-9.95) | 3.85  (2.50-5.93) | 9.74  (8.10-11.71) | 2.42  (1.90-3.08) |
|  | Efficacy % (Mean±SD) | 95±2.56 | 81±6 | 97±1.14 | 67±6 | 96±2.34 |
| Temozolomide | IC50 µM | NR | NR | NR | NR | NR |
|  | Efficacy % (Mean±SD) | 15±5.68 | 10±1.55 | 1±2.30 | 7±6.05 | 35±12.19 |

Abbreviations: CHT, chemoterapeutic; NR, Not Responsive.

**Supplementary Table S5. Proteins of the melanosome cellular component from STRING functional enrichment analysis up- and down-regulated in SN-MM5 compared to other cell lines.**

|  | **SN-MM5 vs NHEM** | **SN-MM5 vs**  **SN-MM1** | **SN-MM5 vs**  **SN-MM2** | **SN-MM5 vs**  **SN-MM3** | **SN-MM5 vs**  **SN-MM4** |
| --- | --- | --- | --- | --- | --- |
| **Up-regulated** | AHCY, RAB38, CANX, ERP29, PDIA4, CTSD, SDCBP, CAPG, YWHAE, ATP6V1B2, ATP1B3, CTSB, ANXA2, ANXA6, RAB32, MLANA, GPNMB, TYRP1, ATP1A1, CD63 | SYPL1, HCY, LC1A4, RAB38, SERPINF1, ERP29, CAPG, STOM, FASN, HSP90AA1, ANXA6, TFRC, RAB32, SLC3A2, MLANA, CCT4, RAB27A, PMEL, CD63 | AHCY, CTSD, RAB38, CANX, ERP29, STOM, HSP90B1, PPIB, PDIA3, P4HB, ANXA6, TFRC, RAB32, RAB27A, PMEL, CD63, SEC22B | AHCY, SLC1A4, CTSD, RAB38, CANX, SDCBP, ERP29, RAB7A, ATP6V1B2, PDIA4, ATP1B3, STOM, RPN1, HSP90B1, PPIB, PDIA3, TMED10, FASN, P4HB, LAMP1, HSP90AA1, CTSB, ANXA6, TFRC, RAB32, SLC3A2, MLANA, RAB27A, PDIA6, RAB1A, PMEL, TMEM33, RAB5C, CD63, SEC22B | AHCY, SLC1A4, CTSD, RAB38, ERP29, RAB7A, ATP1B3, HSP90B1, PPIB, PDIA3, TMED10, FASN, P4HB, LAMP1, TFRC, RAB32, MLANA, GPNMB, RAB27A, PDIA6, RAB1A, PMEL, TMEM33, ATP1A1, RAN, RAB5C, CD63, SEC22B |
| **Down-regulated** | AHCY, RAB38, CANX, ERP29, PDIA4, HSP90B1, TMED10, FASN, P4HB, HSP90AA1, TFRC, RAB27A, PDIA6, TMEM33, NAP1L1 | CTSD, RAB2A, GANAB, ANXA2, ITGB1, CALU, CLTC | SLC1A4, RAB2A, CAPG, SYNGR1, ANXA2, SND1, CALU | SERPINF1, CAPG, ANXA2, ITGB1, DCT, SLC2A1 | RAB2A, YWHAE, ANXA2, SND1, YWHAZ, ITGB1, CALU |
