# Supplementary material for: Novel cellular systems unveil mucosal melanoma initiating cells and a role for PI3K/Akt/mTOR pathway in mucosal melanoma fitness
Source: J Transl Med. 2024 Jan 8;22:35. doi: 10.1186/s12967-023-04784-2 (PMC10775657; doi:10.1186/s12967-023-04784-2)

Supplementary Figures

**FIGURE S1**

**
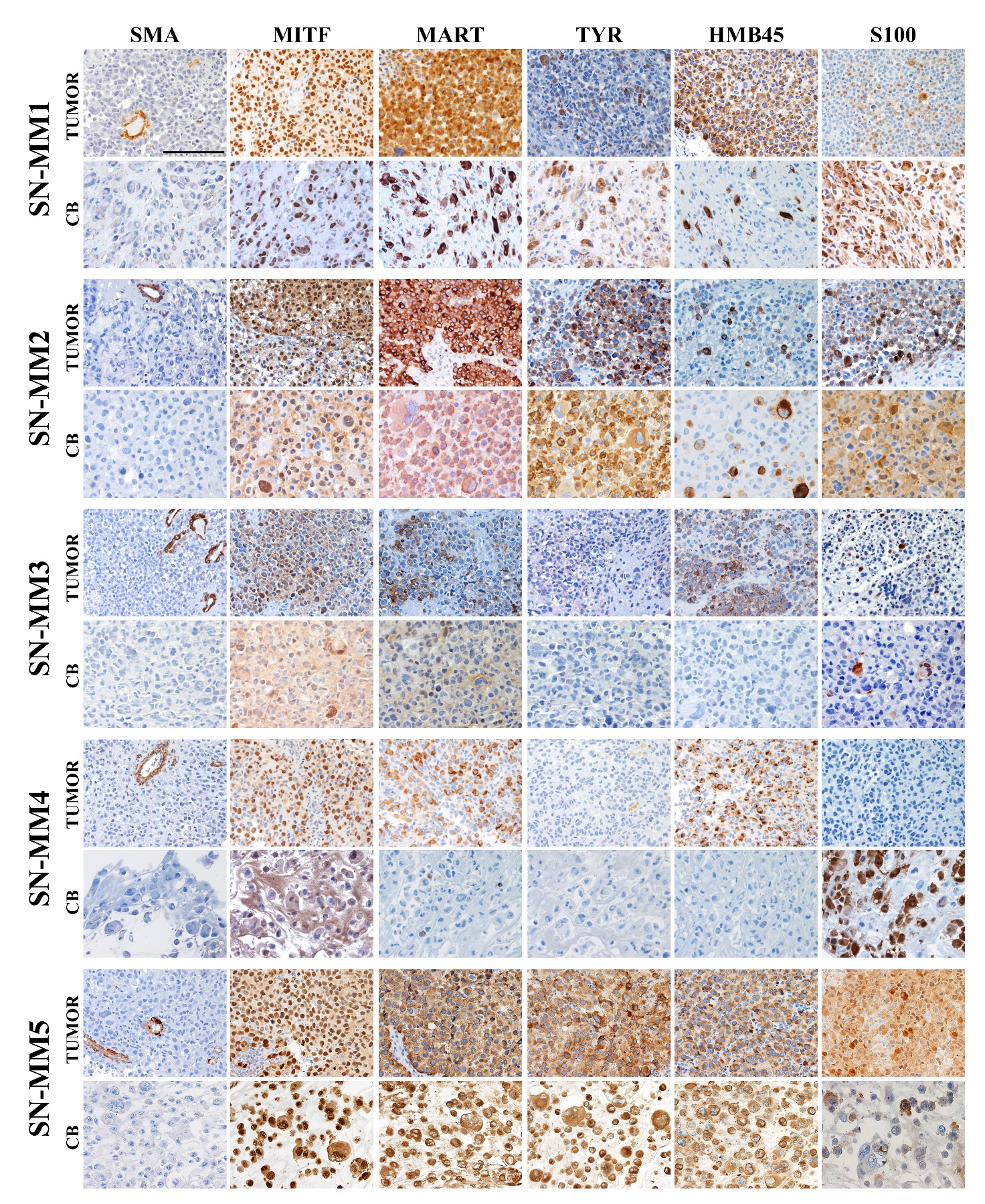
**

**FIGURE S2**

**
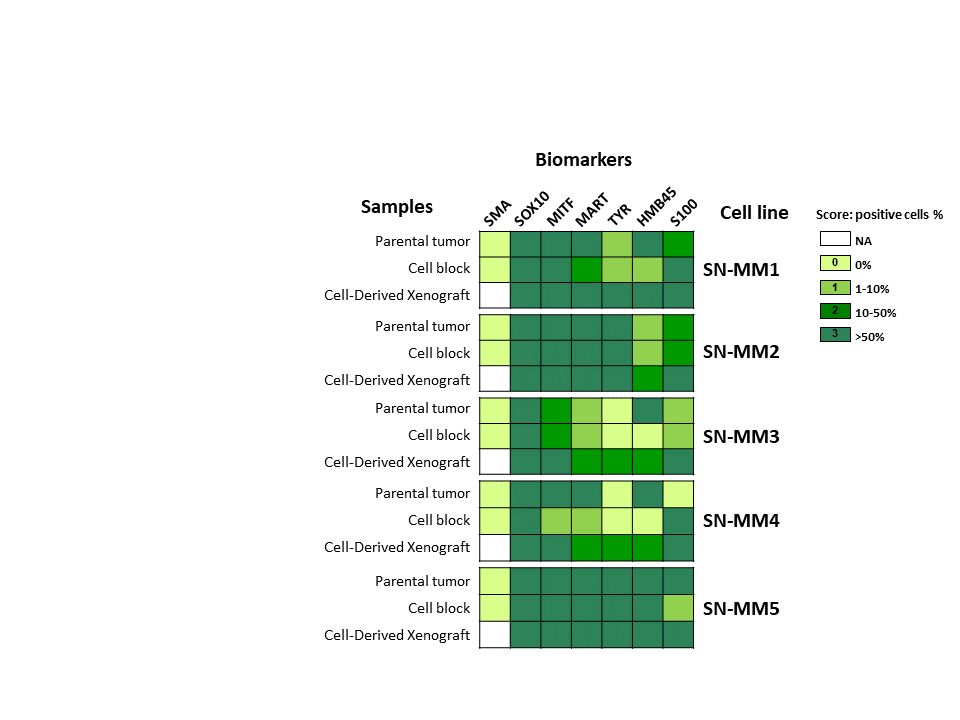
**

**FIGURE S3**

**
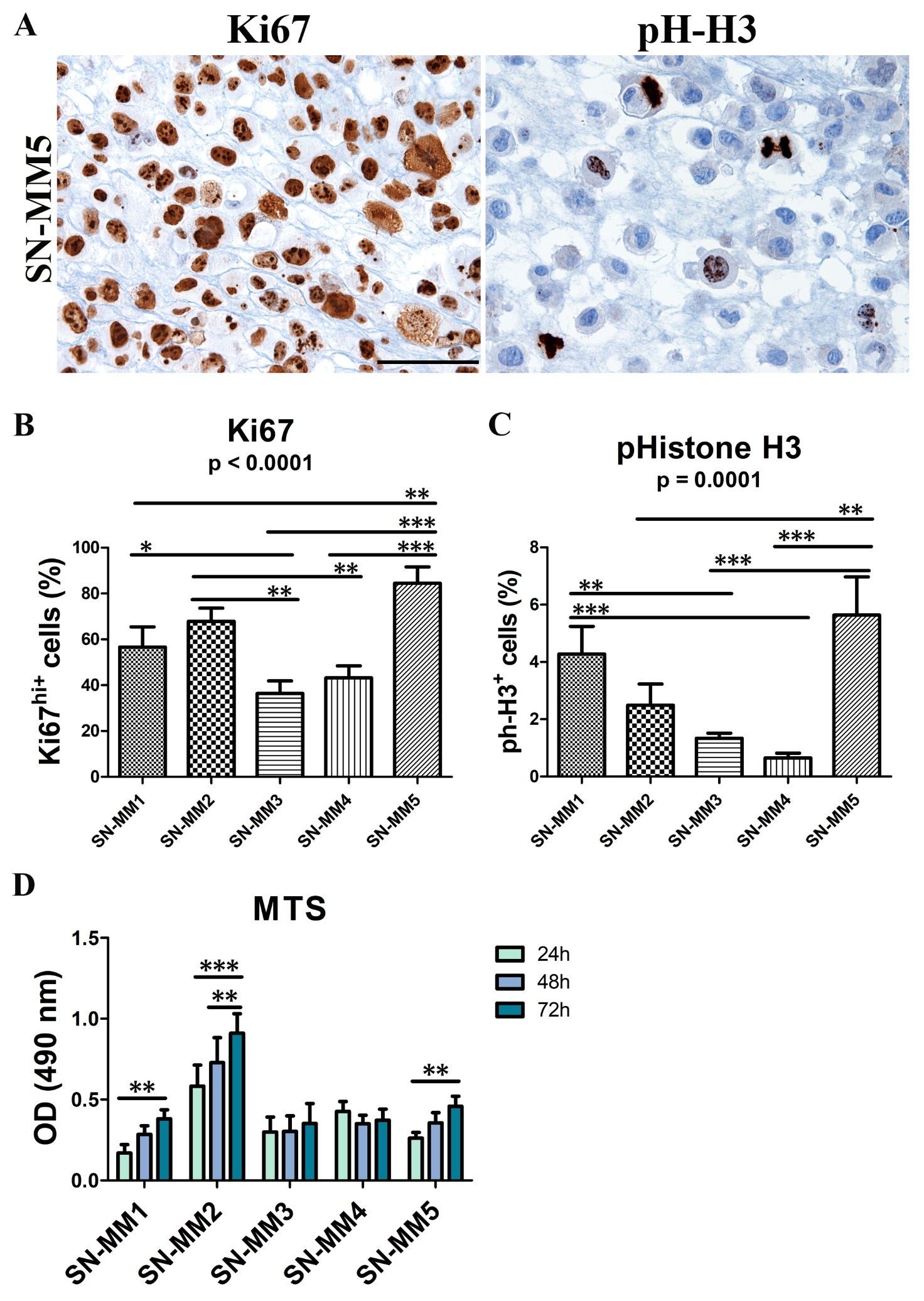
**

**FIGURE S4**

**
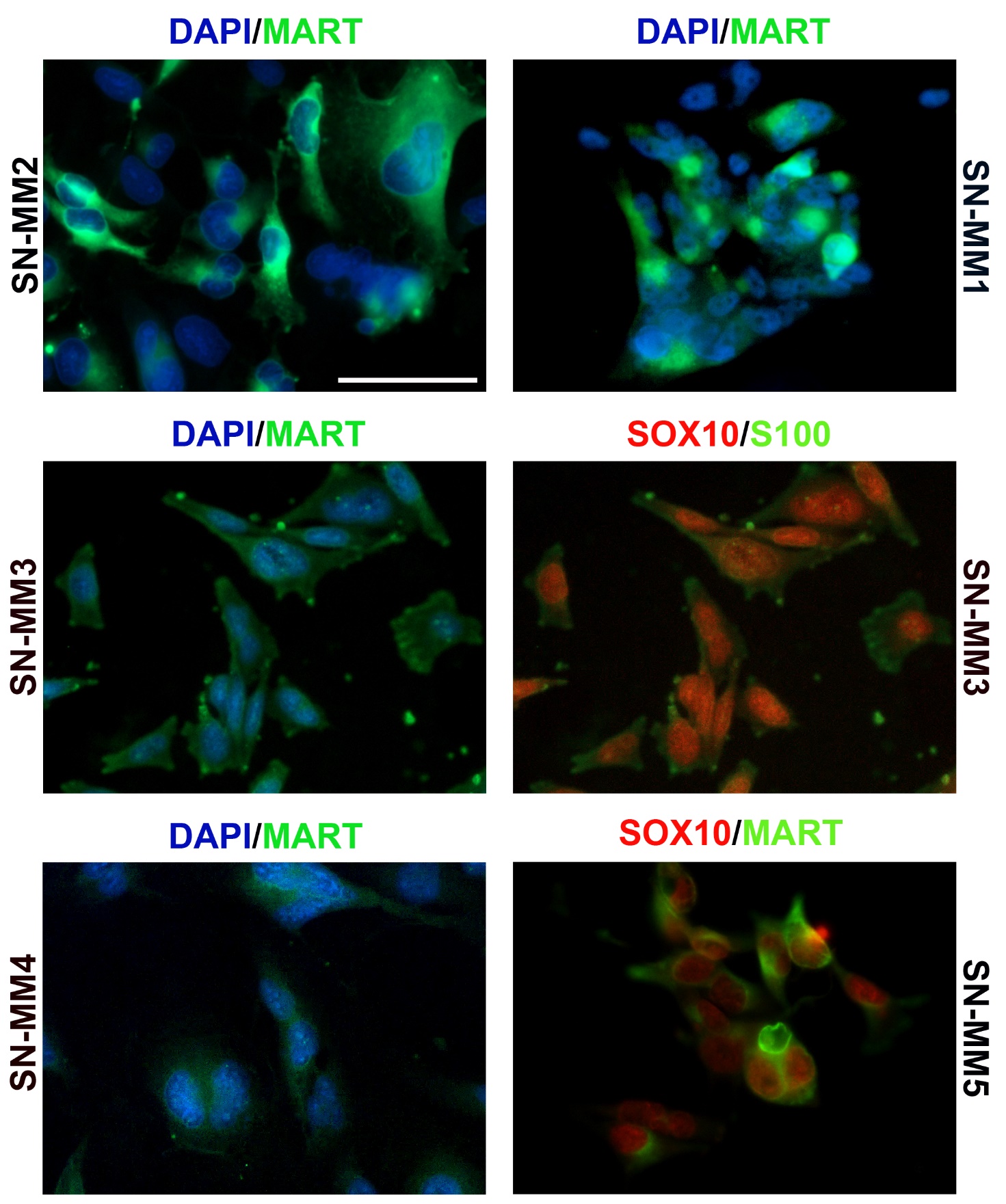
**

**FIGURE S5**
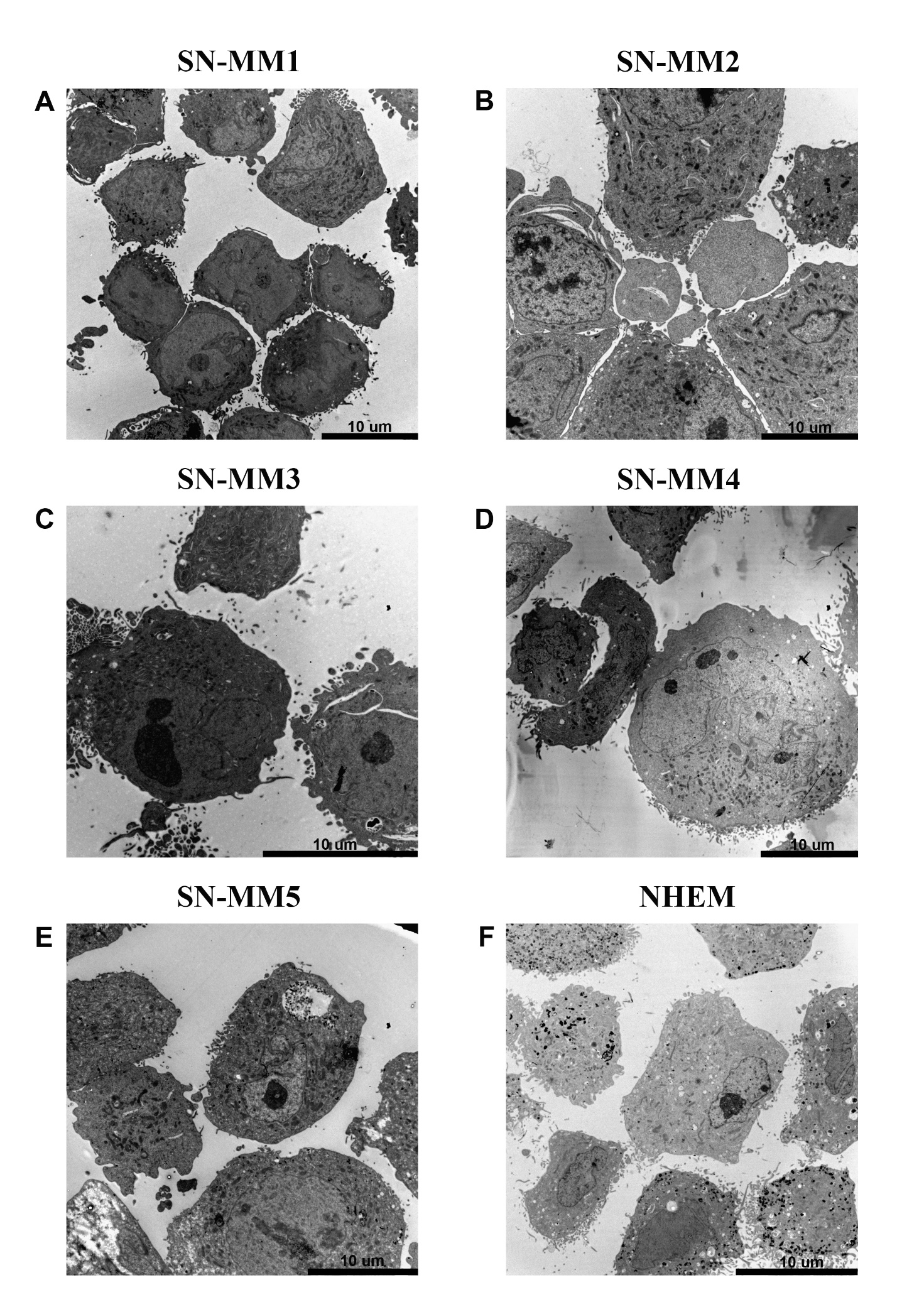


**FIGURE S6**

**
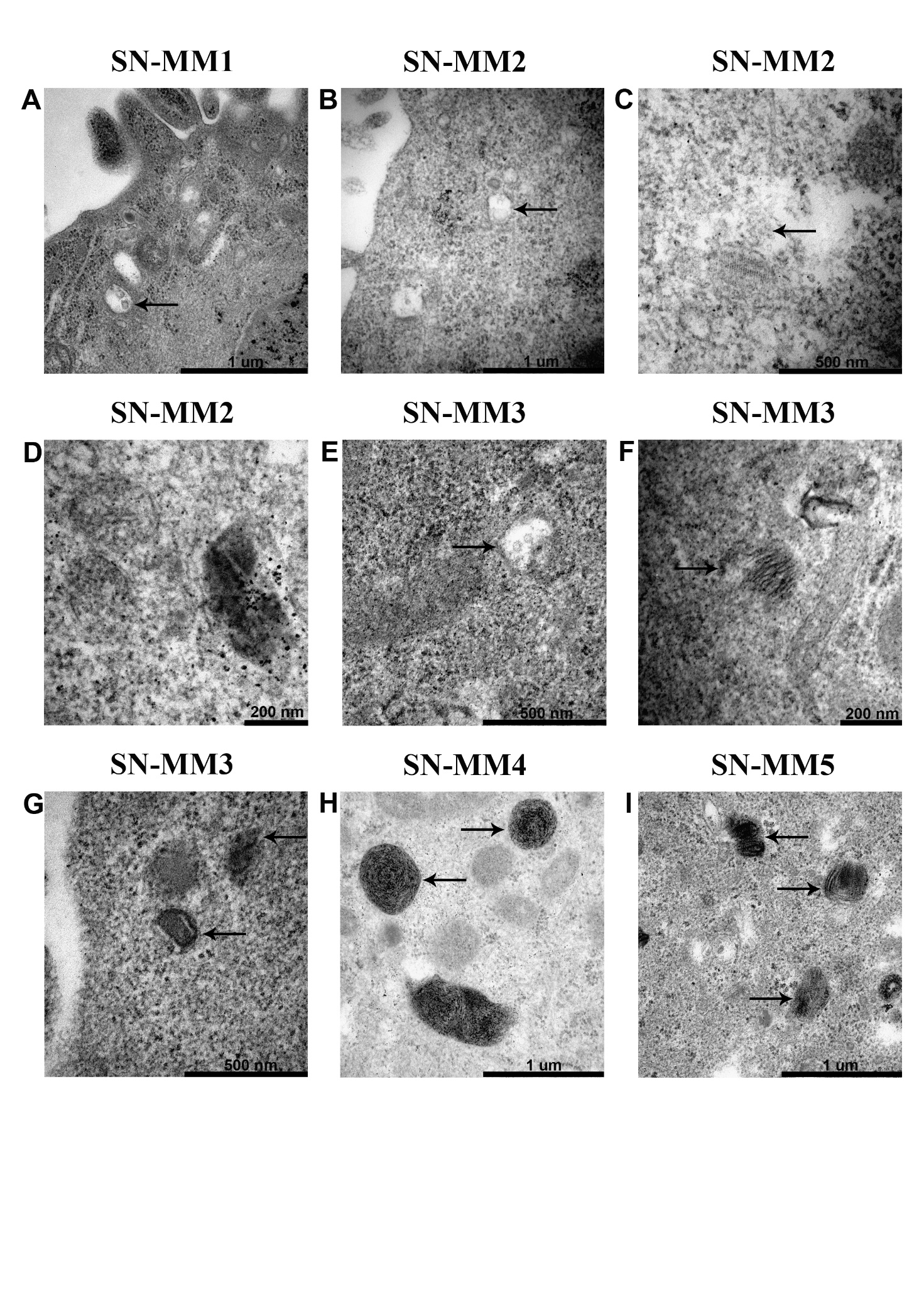
**

**FIGURE S7**

**
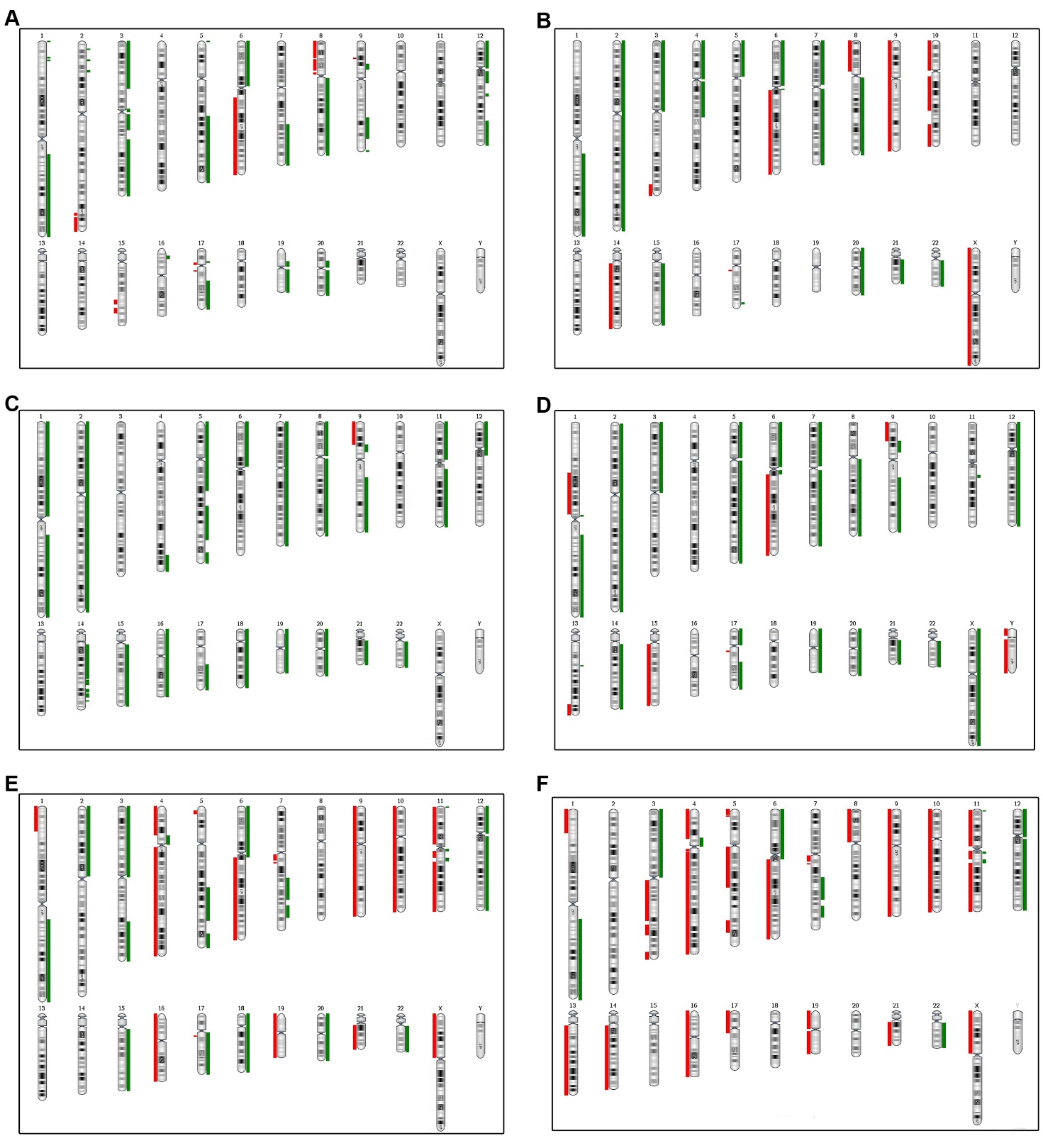
**

**FIGURE S8**

**
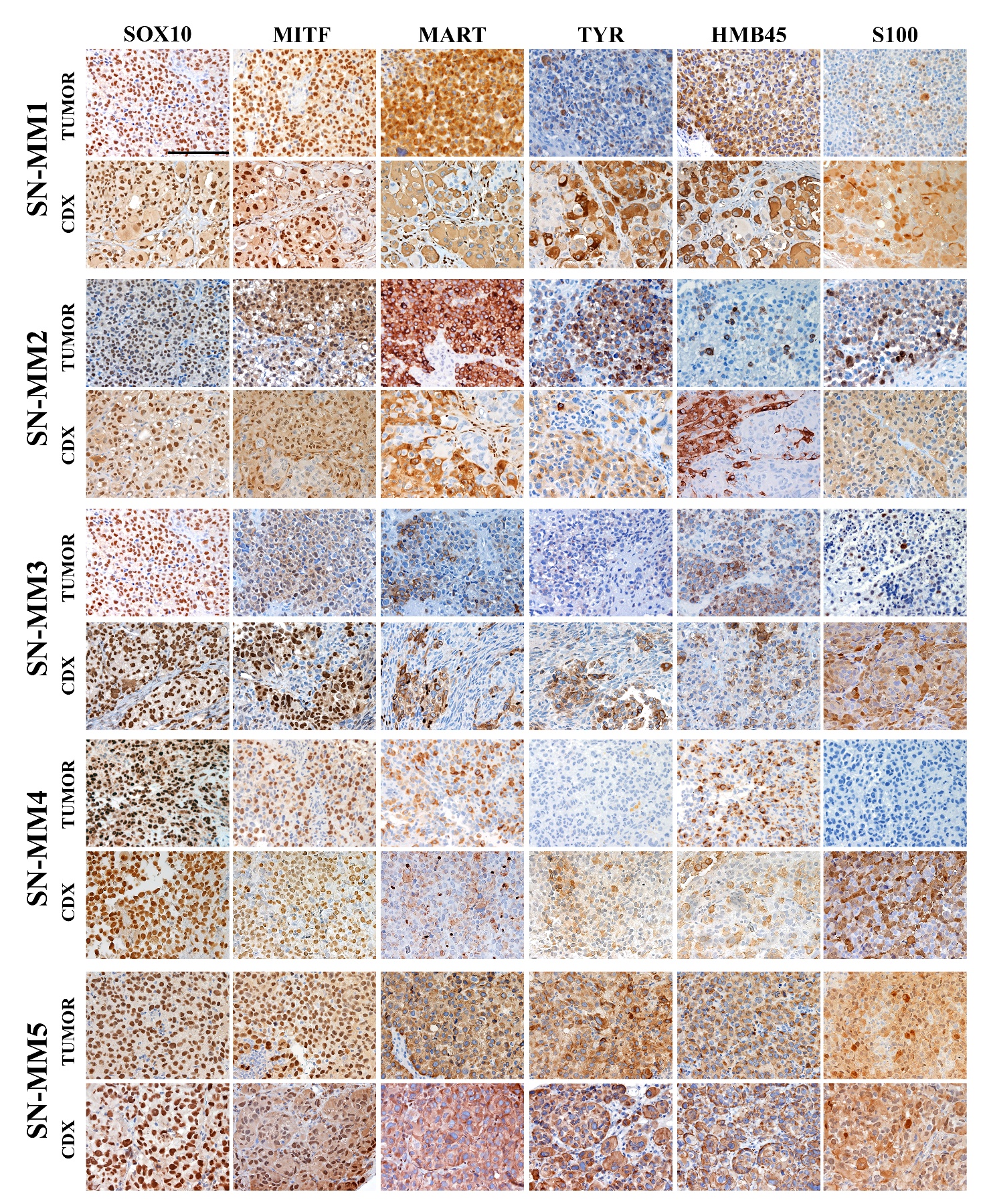
**

**FIGURE S9**

**
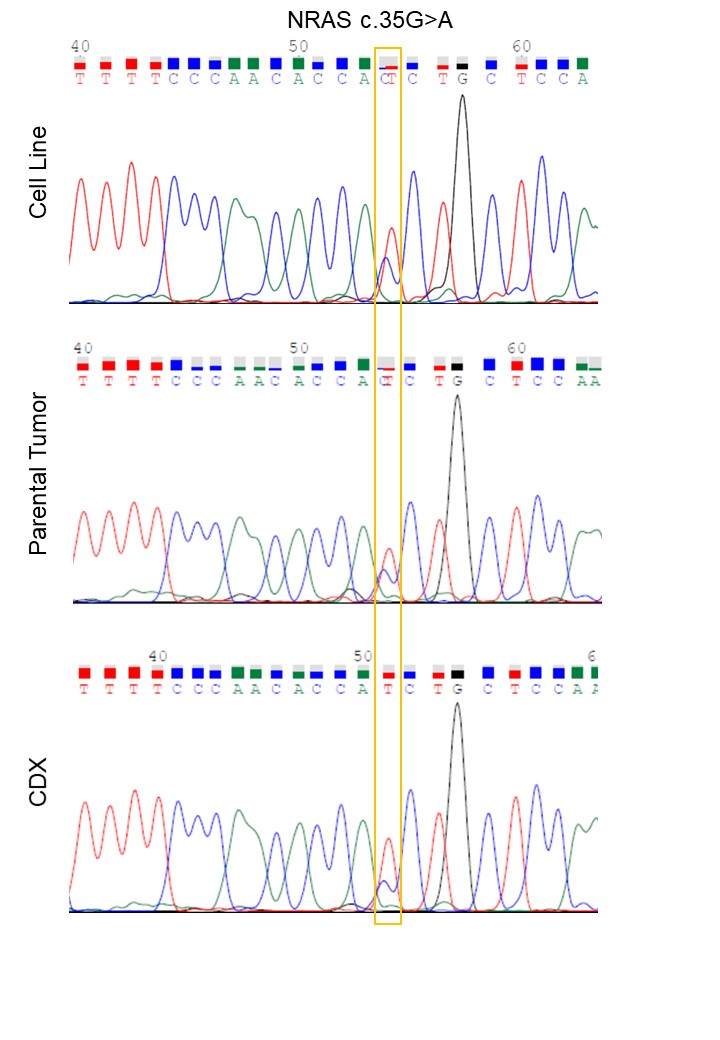
**

**FIGURE S10
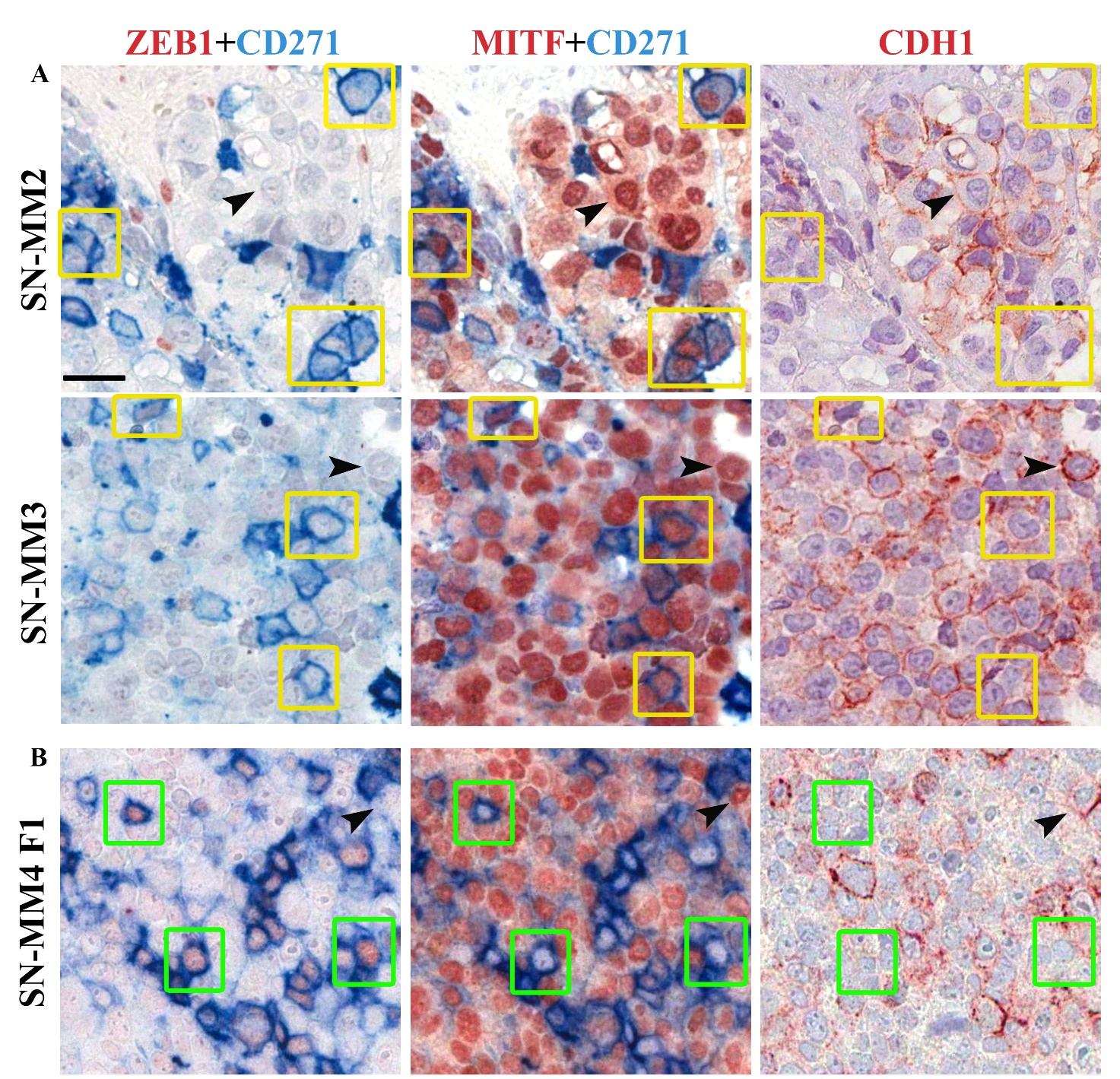
**

**FIGURE S11**

**
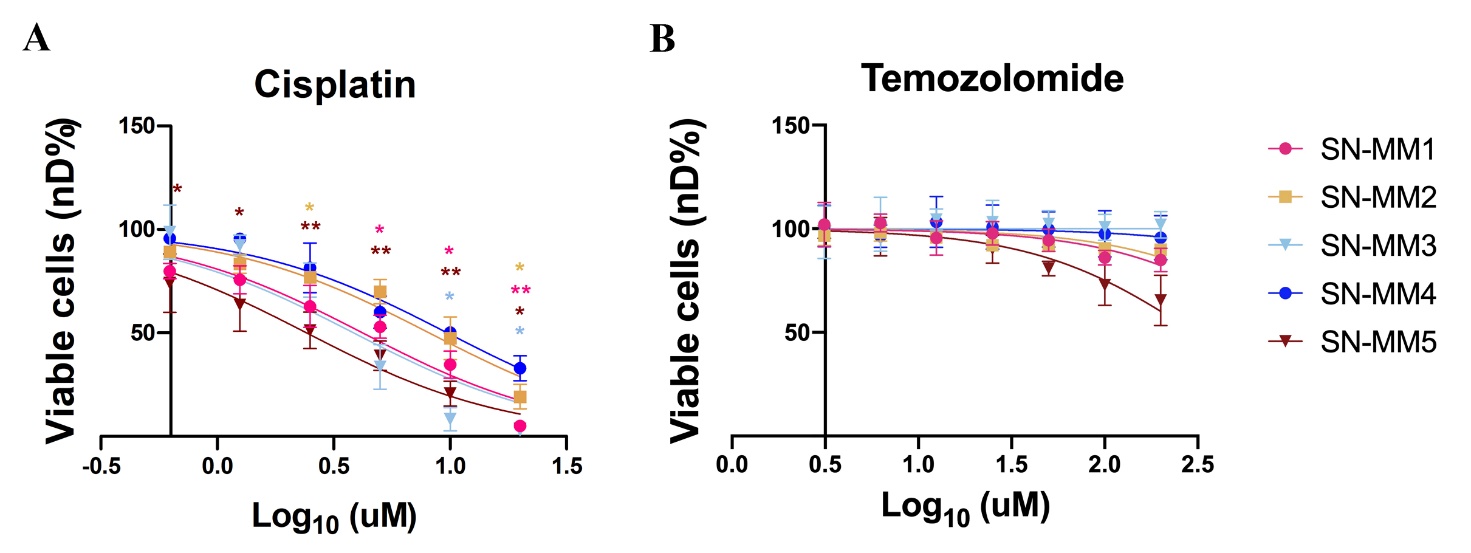
**

**FIGURE S12**

**
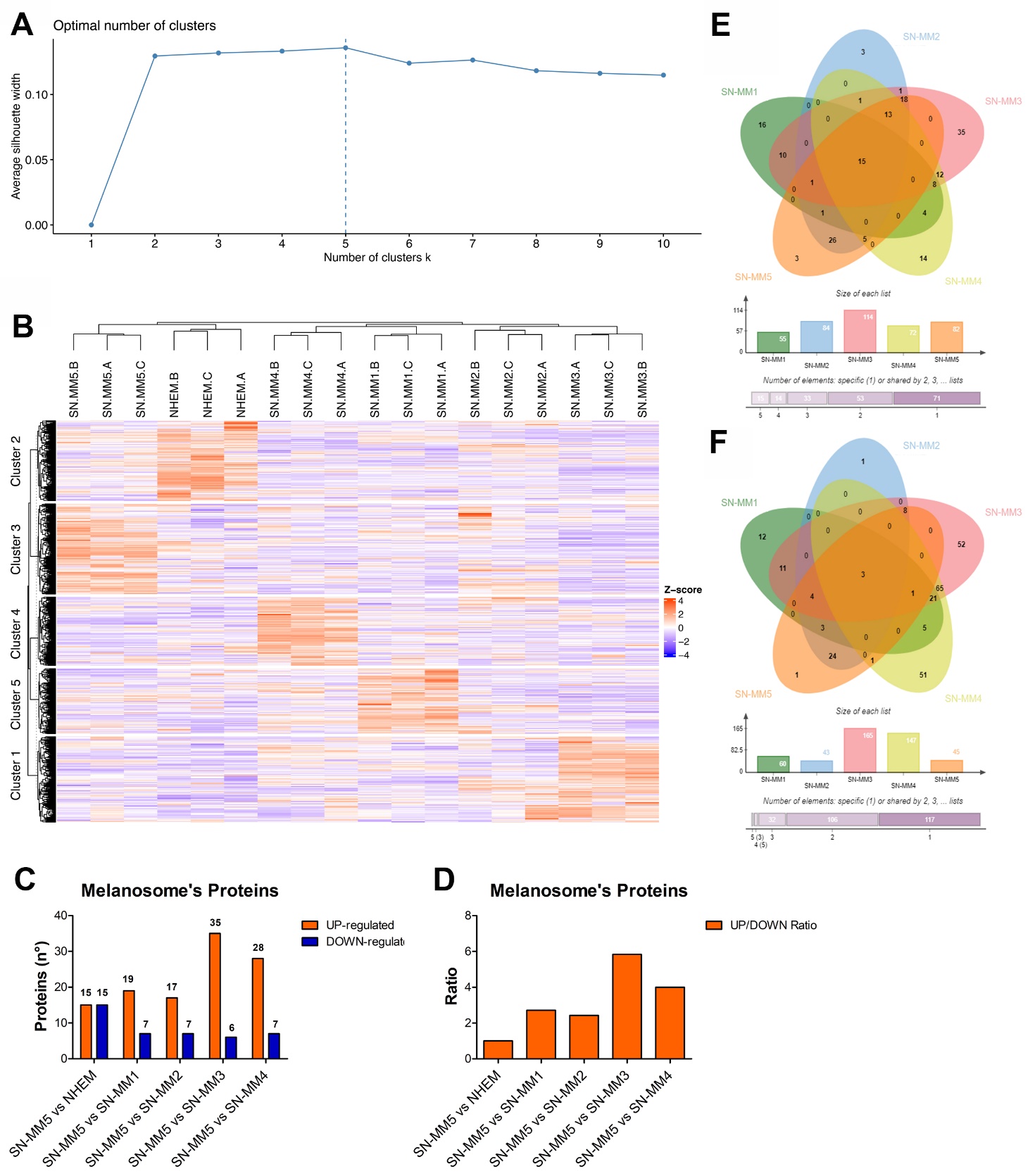
**

**FIGURE S13**

**
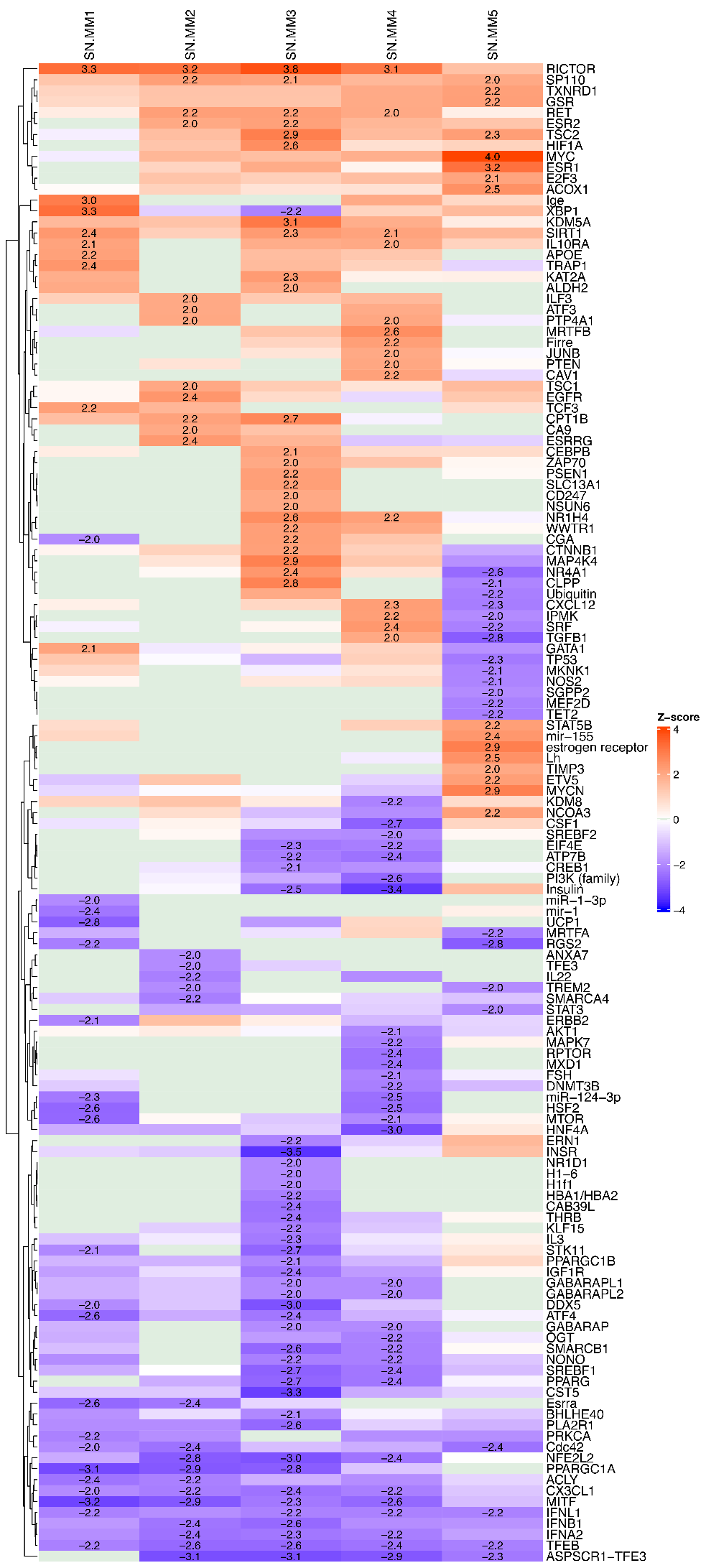
**

**FIGURE S14**


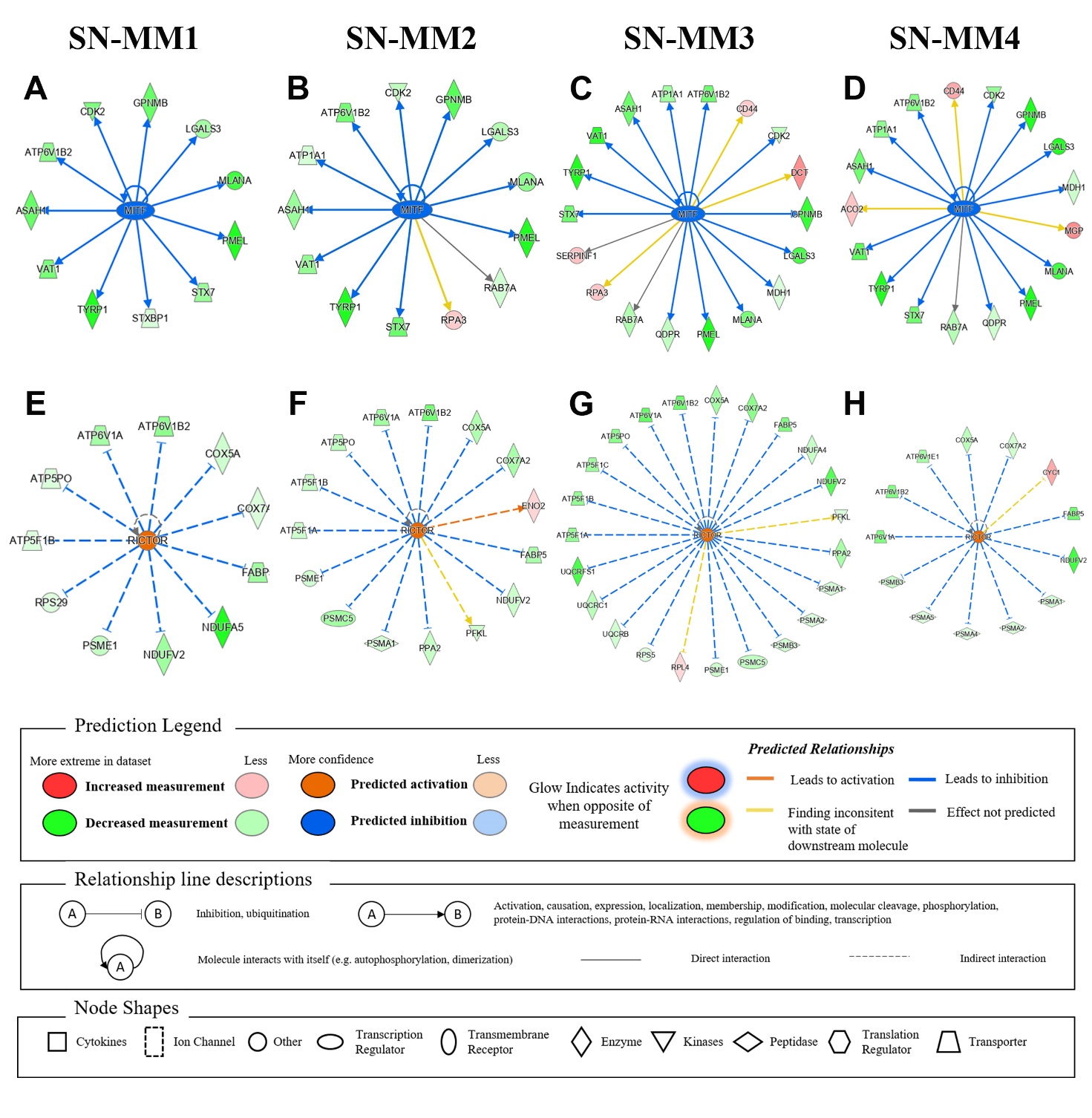


**FIGURE S15**


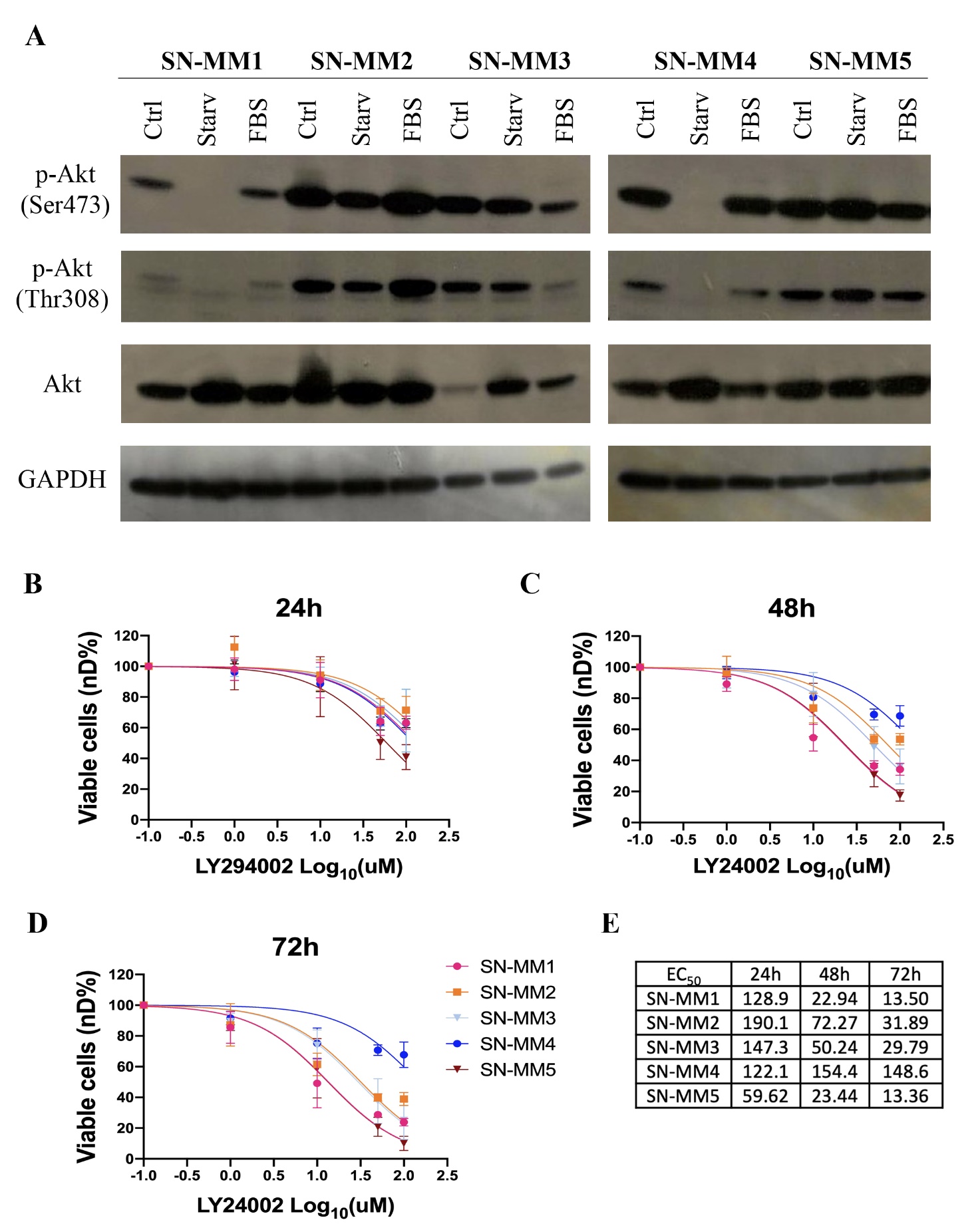

Supplement: Supplementary file 7 — Additional file 7: Figure S1. IHC analysis of SN-MM parental tumor biopsies and corresponding cell blocks (CB). Expression of melanocytic markers MITF, HMB45, MART, TYROSINASE, S100 and tumor associated fibroblast marker α-SMA. Sections are counterstained with hematoxylin. Parental tumors: Magnification 200X, scale bar 100 µm. CB: Magnification 400X, scale bar 50 µm. Figure S2. Scoring of melanocytic biomarkers in patient’s tumors, their corresponding SN-MM cell lines and cell-derived xenografts. Heatmap summarizing immunohistochemical expression of melanocytic biomarkers and α-SMA on the SN-MM cell lines cell block, patient’s tumor biopsies and mouse skin xenografts. Four-tiered scoring system was adopted, as described in methods section. NA: not assessed. Figure S3. Proliferation index and density of mitotic cells in SN-MM cell lines. A Representative images of ki67 and ph-H3 staining in SN-MM5 cell-block. Sections are counterstained with hematoxylin. Magnification 400X, scale bar 50 µm. B, C Quantification of ki67 and ph-H3 staining in SN-MM cell blocks. Histograms represent the percentage of positive cells out of total cells in three 20X field analyzed on digitalized slides. D Proliferation of SN-MM cell lines measured by MTS assay at 24 h, 48 h, and 72 h (n = 5). Histograms represent the absorbance at 490 nm that is directly proportional to the number of live cells in culture. One-way ANOVA statistical analysis and Bonferroni’s multiple comparison post-test have been performed. *p < 0.05; ** p < 0.01; *** p < 0.001. Figure S4. Immunofluorescence staining of melanocytic biomarkers in SN-MM cell lines. SN-MM cells were cultured and stained as labeled. Cell nuclei were stained with DAPI (blue). Representative images of expression of MART (green), SOX10 (red) and S100 (green) are shown. Scale bar 20 µm. Figure S5. Ultrastructural features of SN-MM cell lines. TEM images showing cell morphology and heterogeneity among SN-MM cell lines (A–F); electron dense melanocyt [file 12967_2023_4784_MOESM7_ESM.docx]
